# TLR9 activation in large wound induces tissue repair and hair follicle regeneration via γδT cells

**DOI:** 10.1038/s41419-024-06994-y

**Published:** 2024-08-17

**Authors:** Xinhui Li, Tiantian An, Yang Yang, Zhaoyu Xu, Shuaidong Chen, Zumu Yi, Chen Deng, Feng Zhou, Yi Man, Chen Hu

**Affiliations:** 1 State Key Laboratory of Oral Diseases, West China Hospital of Stomatology, Sichuan University, Chengdu, 610041 Sichuan China; 2 Department of Oral Implantology, West China Hospital of Stomatology, Sichuan University, Chengdu, 610041 Sichuan China

**Keywords:** Regeneration, Inflammation, Mucosal immunology

## Abstract

The mechanisms underlying tissue repair in response to damage have been one of main subjects of investigation. Here we leverage the wound-induced hair neogenesis (WIHN) models in adult mice to explore the correlation between degree of damage and the healing process and outcome. The multimodal analysis, in combination with single-cell RNA sequencing help to explore the difference in wounds of gentle and heavy damage degrees, identifying the potential role of toll-like receptor 9 (TLR9) in sensing the injury and regulating the immune reaction by promoting the migration of γδT cells. The TLR9 deficient mice or wounds injected with TLR9 antagonist have greatly impaired healing and lower WIHN levels. Inhibiting the migration of γδT cells or knockout of γδT cells also suppress the wound healing and regeneration, which can’t be rescued by TLR9agonist. Finally, the amphiregulin (AREG) is shown as one of most important effectors secreted by γδT cells and keratinocytes both in silicon or in the laboratory, whose expression influences WIHN levels and the expression of stem cell markers. In total, our findings reveal a previously unrecognized role for TLR9 in sensing skin injury and influencing the tissue repair and regeneration by modulation of the migration of γδT cells, and identify the TLR9-γδT cells-areg axis as new potential targets for enhancing tissue regeneration.

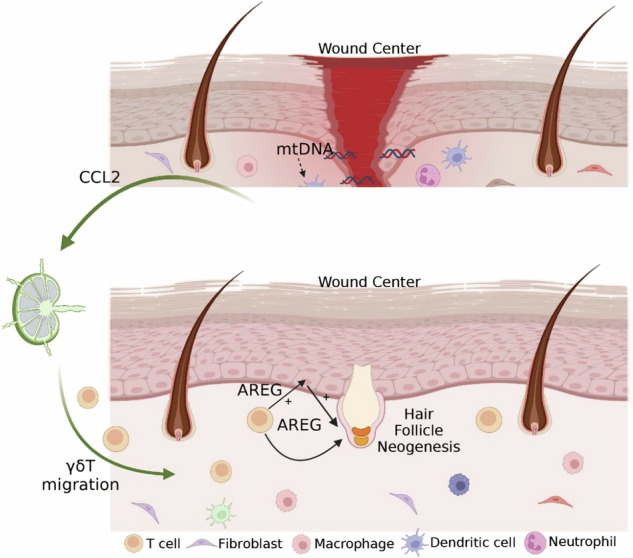

## Introduction

Scar-free healing and functional regeneration after tissue injury have long been desired goals. However, the fundamental logics that regulate the initiation and process of tissue repair and regeneration remains unclear. Damage associated molecular patterns (DAMPs) serve as central players of signaling molecules that indicate tissue injury, and involve in the initiating of inflammation and regenerative processes [1].

The intricate signaling transduction pathway between damage-associated molecular patterns (DAMPs) and Toll-Like receptor 2 (TLR2) has shown to stimulate the proliferation and differentiation of renal tubular progenitor cells, thereby facilitating the regeneration and repair of renal tubules [2]. However, advancing research on various types of DAMPs and pattern recognition receptors (PPRs) has revealed that their involvement in tissue repair or regeneration is not invariably advantageous. Excessive responses triggered by DAMPs have been implicated in exacerbating left ventricular remodeling post-myocardial infarction, leading to inflammatory infiltration extending beyond the infarcted myocardium, activation of pro-apoptotic pathways, further loss of myocardial cells, escalated matrix degradation, compromised collagen deposition, and the promotion of scar formation [3].

Skin and its appendages have served as pivotal model systems in regenerative medicine due to their remarkable regenerative capacity, often yielding novel insights and principles. While conventional wisdom suggests that skin wounds primarily heal through regeneration during embryonic or neonatal stages, a remarkable ability for hair follicle (HF) regeneration in adult mice and rabbits has been observed in substantial full-thickness wound centers within the context of wound-induced hair follicle neogenesis (WIHN) models [4, 5]. Notably, WIHN exclusively manifests in sufficiently large wounds, with small wounds failing to elicit regenerative responses. This phenomenon prompts contemplation on the interplay between pathogen-associated molecular patterns (PAMPs), inflammatory cascades, and regenerative outcomes. It raises the question of whether the generation of hair follicles in WIHN wounds stems from more extensive skin damage, resulting in heightened release of DAMPs and activation of PPRs. The precise mechanisms underlying how these factors modulate immune and reparative responses, impact stem cell fate, and influence hair follicle neogenesis remain incompletely elucidated.

While some literature suggests that excessive or prolonged inflammatory reactions may culminate in fibrosis [6–8], others propose that a robust regenerative response can be sparked by heightened inflammatory signals [9, 10]. Hence, it beckons exploration into how the interplay of DAMPs and PPRs with inflammatory responses alters under conditions of exacerbated tissue damage and the subsequent impact of this heightened inflammatory milieu on stem cells and other cells mobilization.

Our prior investigations have demonstrated that the introduction of aligned extracellular matrix (ECM) scaffold yields enhanced regeneration of hair follicles, concomitant with an immunomodulatory effect [11, 12]. Additionally, our another study has delved into the essential role played by adaptive immune cells in the process of wound healing and regeneration [12]. However, further research is warranted to unravel how diverse immune cells respond to signals emanating from tissue damage, initiating and coordinating tissue repair and particularly regeneration-related responses. To mitigate the potential confounding factors arising from variations in wound sizes when comparing large and small wounds, we crafted several wound models with equated surface areas but differing degrees of tissue damage.

We firstly revealed the association between the extent of tissue damage with the number of regenerated hair follicles and the expression levels of Toll-like Receptor 9 (TLR9). The role of TLR9 in mediating the correlation between degrees of injury and WIHN levels was investigated by comparing mice with enhanced or repressed activation levels of TLR9 or knockout of TLR9. Then the single cell RNA sequencing, were conducted and revealed the comprehensive immune reaction changes under the enhanced activation of TLR9. Of note, the number of γδT cells greated elevated with higher expression of pertinent chemokines in TLR9 activated wounds. Inhibiting the migration or knockout of TCRδ eliminated the function of TLR9. Finally, we also figured out the effector Areg, secreted by γδT and keratinocytes, played a role in promoting the expression of stem cell markers and regeneration markers including Twist1, Wnt7b, Ctnnb1 and so on. Collectively, our study delineates a comprehensive process and offers fresh perspectives on how varying levels of damage-associated molecular patterns modulate the immune response through their interaction with PPRs, ultimately shaping the behavior of stem cells pertinent to regeneration.

## Results

### Hair follicle neogenesis was positively correlated with extent of tissue damage, and this process was dependent on TLR9

**Fig. 1:**
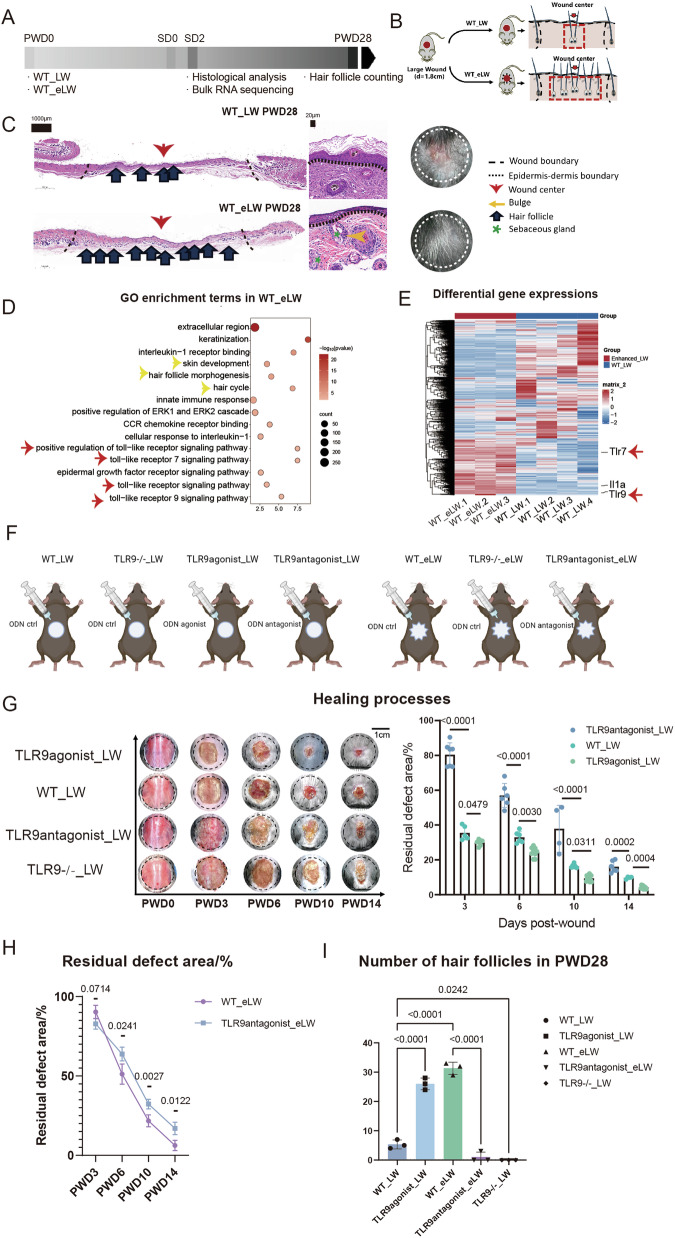
The differential regeneration results in WT_eLW and the effects of TLR9 in this process. **A** Workflow for evaluating large-scale wound healing. **B** Surgical processes for skin wound models. **C** Representative H&E images and appearances of WT_LW and WT_eLW at PWD28. **D** GO enrichement terms up-regulated in WT_eLW compared with WT_LW. Yellow arrows are terms related with skin and hair follicle development. Red arrows are terms related with toll-like receptor signaling pathway. **E** The differential expressed genes between WT_LW and WT_eLW. **F** The different types of skin wounds and reagents dropwise added in wounds. **G** The healing speed and residual wound area (%) in groups of standard wounds. Wound were treated with TLR9agonist (ODN2395, Invivogene, 4 μg per time per mouse) at PWD 0,3,6 (TLR9agonist_LW); Ctrl ODN (ODN2395 control, Invivogene, 4 μg per time per mouse) (WT_LW); TLR9antagonist (TLR9antagonist_LW) (ODN2088, Invivogene, 4 μg per time per mouse); or wound in TLR9 −/− mice treated with Ctrl ODN (ODN2088 control, Invivogene, 4 μg per time per mouse) (TLR9 −/−_LW) respectively. *n* = 4–8 for each group. **H** The residual defects area (%) in enhanced wounds. Wounds were treated with Ctrl ODN (ODN2088 control, Invivogene, 4 μg per time per mouse) (WT_eLW); or TLR9antagonist (TLR9antagonist_eLW) (ODN2088, Invivogene, 4 μg per time per mouse) at PWD0,3,6 respectively. *n* = 4–8 for each group. **I** The number of regenerated hair follicles in PWD28. *n* = 3 for each group. All error bars ±SD.

We refer to the classic WIHN model, which involves creating a large wound on the back of mice and allowing it to heal naturally without splinting. In order to trigger and explore signals associated with tissue damage as strongly as possible, we enlarged the diameter of the wound to 1.8 cm. To eliminate the asynchrony of wound healing caused by different healing speed under different situations, we used the second day after re-epithelialization (SD2) as a detection window phase for hair follicle regeneration, as hair follicles begin to form at around SD 0-7 days reported in relevant literatures (Fig. 1A) [13, 14]. To investigate the impact of wound severity on regeneration outcomes, we made the enhanced injury on large wound (WT_eLW) model in study of Nelson et al. [15] by adding 6–8 short radial incisions around the circular wound, which increased the degree of wound damage without changing the wound area. Since many studies suggest that many factors including biomechanical forces/tension and tissue electric current etc. may also affect the degree of regeneration [16], we designed three different types of enhanced large wound models to reduce the interference of these confounding factors (Fig. S1A). By comparing various wound types, we found multiple types of wounds could enhance hair follicle regeneration compared with standard large wounds (WT_LW) at 28 days post wounded (PWD28) (Fig. S1B).

To further explore the relationship between injury severity and hair follicles, and to dissect the biological effects of enhanced injury on the skin, we collected the tissues at wound center of WT_eLW and WT_LW at SD2 and conducted bulk-RNA sequencing (bulk-RNA seq) (Figs. 1A and S1C).The gene ontology (GO) enrichment analysis showed that multiple TLRs family pathways were upregulated in WT_eLW compared with WT_LW, especially TLR7 and TLR9, as observed by qRT-PCR (Figs. 1D, E and S1D–G).

Toll-like receptor (TLR) family, as one of the important pattern recognition receptor families in the in–nate immune system, has long been believed to play a role in sensing infections and tissue damage. Members of the TLR family such as TLR3, 7, and 9 can be activated by signals related to tissue damage [17]. Therefore, based on the significant upregulation of the Toll-like receptor family in RNA-seq data, we speculate that greater tissue damage promotes the activation of the Toll-like receptor family. Additionally, GO enrichment analysis suggests that the TLR7 and TLR9 signaling pathways are the most significantly upregulated TLR signaling pathways. Overall, there have been numerous studies investigating the role of TLR7 in skin wounds, inflammation, and development because of its significant role in various skin inflammations, allergies, autoimmune diseases and so on [18, 19]. However, research on the role of TLR9 in skin, especially in wound repair and regeneration, is rare. Some studies have found that TLR9 is upregulated after tissue damage and promotes wound repair [20]; however, other studies suggest that TLR9 may promote the transformation of fibroblasts into myofibroblasts, leading to fibrosis [21, 22]. Therefore, overall, there is less research on TLR9, and conclusions about its characteristics are not yet clear. How the activation of TLR9 affects the wound healing process, especially in the late stage, and whether it can influence the immune microenvironment to control the reactivation of embryonic-like programs related to hair follicle development, remains unknown.

To explore the effects of TLR9 on wound healing and HF regeneration, we dropwise added the TLR9 agonist ODN 2395 (4 μg per injection, InvivoGen, America) or the TLR9 antagonist ODN 2088 (4 μg per injection, InvivoGen, America) and TLR9^−/−^ mice (C57BL/6Smoc-Tlr9em1Smoc) (Cat. NO. NM-KO-190168, Shanghai Southern Model Biological Co., LTD) to compare the healing speed and regeneration results. The workflow for evaluating large wound healing is summarized in Fig. 1F. The TLR9 agonist significantly accelerated the healing speed, while inhibiting or knock out of TLR9 dampened wound healing greatly both in WT_LW or WT_eLW (Fig. 1G, H). In addition, the number of hair follicles in PWD28 was increased by 3.6 times in TLR9 activated standard wounds (TLR9agonist_LW) and 3.9 times in wounds with enhanced damage (WT_eLW) (Fig. 1I). The injection of TLR9 antagonist eliminated the enhanced hair follicle regeneration in WT_eLW (Fig. 1I), indicating the positive correlation between hair follicle regeneration with extent of tissue damage was dependent on TLR9. Therefore, we inferred that TLR9 might play an essential role in wound healing and HF regeneration in large wounds as a wound-related sentinel.

### The activation of TLR9 was from the enhanced release of mtDNA in wounds with larger extent of damage

**Fig. 2:**
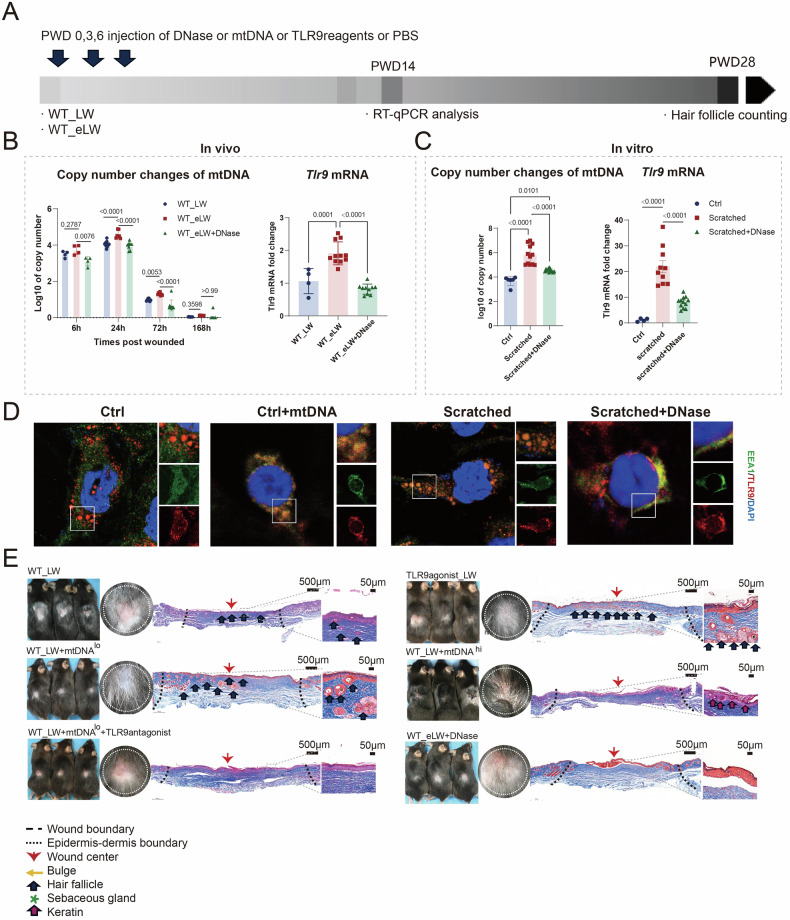
The injury activated TLR9 by promoting release of mtDNA. **A** Workflow for evaluating the release of mtDNA and activation of TLR9 in vivo. **B** The in vivo changes of copy numbers of mtDNA in plasma of mice and qRT-PCR mRNA expression changes of Tlr9 in standard (WT_LW) or enhanced_injury wounds (WT_eLW) and enhanced_injury wounds rejected with DNase I (15 units) (WT_eLW+DNase) at PWD 0, 3, 6. The qRT-PCR of Tlr9 was conducted in tissues collected at PWD14 of the wound center. **C** The in vitro detect of copy numbers of mtDNA and qRT-PCR mRNA fold changes at 24 h after being scratched. Supernate from control M0-THP1 (treated with 100 ng/ml PMA for 24 h) and M0-THP1 with 8 scratches per well in six-well plates (scratched) and scratched cells incubated with 1 μg/ml DNase I (scratched + DNase) were collected to detect the copy numbers of mtDNA. **D** The immunofluorescent staining of TLR9 (red), EEA1 (green) and DAPI (blue) in control M0-THP1 (treated with 100 ng/ml PMA for 24 h), control + mtDNA M0-THP1 (treated with 100 ng/ml PMA for 24 h and then added with 100 ng/ml mtDNA for 24 h), M0-THP1 with 8 scratches per well in six-well plates (scratched) and scratched cells incubated with 1 μg/ml DNase I (scratched + DNase). **E** The Masson staining sections and appearance of wounds for every group. The photos and sections were collected at PWD28. *n* = 3 for each group. All error bars ±SD.

After we proved the effects of TLR9 activation in wound healing, we want to figure out the sources of enhanced expression of TLR9 in WT-eLW. TLR9, as one of the main nucleic acid-sensing toll-like receptors, discovered to recognize unmethylated CpG DNA, typical of bacterial DNA and mtDNA [23]. MtDNA, being structurally analogous to bacterial DNA, is characterized by the presence of numerous unmethylated DNA regions referred to as CpG islands. Mitochondrial DNA is generally known as an immune stimulatory molecule and is released from necrotic cells after traumatic injury or surgery to activate TLR9, stimulator of interferon genes (STING) and so forth [24]. Therefore, we guessed that larger injuries might cause stronger release of mtDNA, which affected the activation of TLR9. To test this speculation, we firstly detected the copy numbers of mtDNA in plasma of WT_LW and WT_eLW mice and found higher copy numbers of Cytochrome B (CytB) in mtDNA in larger extent of wounds (Fig. 2B), accompanied by a higher Tlr9 mRNA in PWD14 (Fig. 2B).

To more intuitively study the association between cellular damage, mtDNA, and TLR9, we simulated tissue damage through scratch experiments using adherent THP-1 cells induced by phorbol ester (PMA). We found that the copy numbers of cytochrome b (CytB) and the mRNA levels of Tlr9 increased after 24 h of cell scratch (Fig. 2C). TLR9 is usually located in the endoplasmic reticulum of the cytoplasm and then transferred to endosomes after sensing of dsDNA [25]. Therefore, we performed immunofluorescent (IF) staining on TLR9 and the marker of endosomes, EEA1, and found that TLR9 was co-localized with EEA1 after scratch-induced cellular damage. In addition, the process was dependent on mtDNA (Fig. 2D).

To prove mtDNA affected hair follicle regeneration through TLR9, different levels of mtDNA extracted from mouse liver was injected into mice through the vein, and at the same time the DNase I was injected into wounds at PWD0,3,6 to ablate mtDNA in another group of mice. Different doses of mtDNA (20 μg (mtDNA^lo^_LW) and 80 μg (mtDNA^hi^_LW) were chosen in this test. We found that the numbers of hair follicles decreased after clearance of mtDNA, while injecting mtDNA into vessels increased hair follicles, which was inhibited by TLR9 antagonist (Fig. 2E).

### The immune changes caused by TLR9 activation on large wound healing and HF regeneration was investigated by single-cell RNA sequencing

**Fig. 3:**
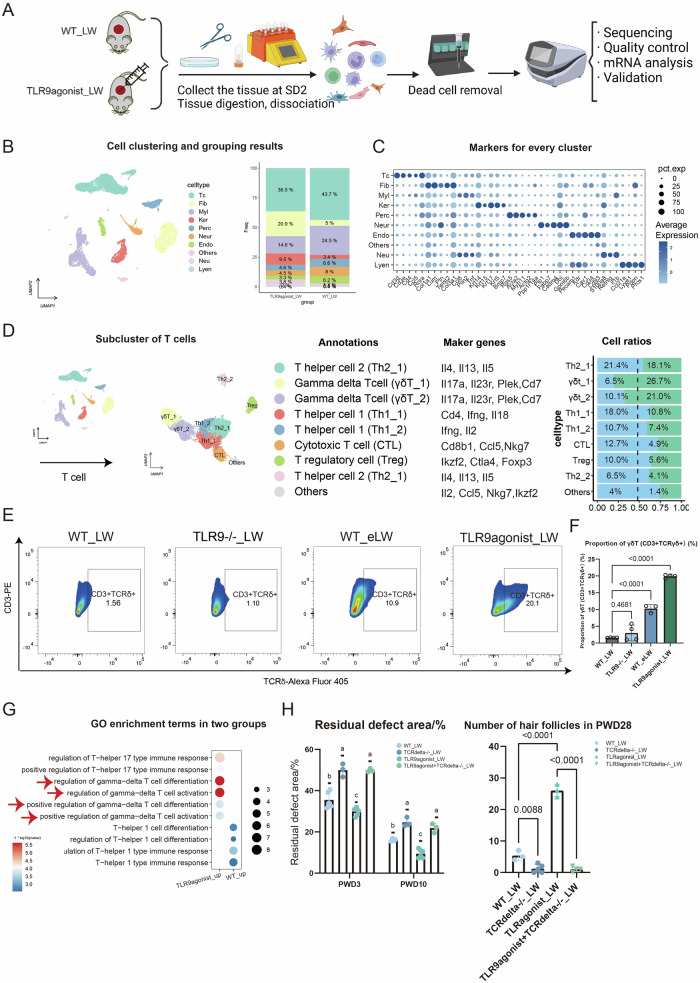
The single-cell atlas of the TLR9 activated microenvironment. **A** Schematic for generating scRNA-seq data from large area excisional wounds on SD2. **B** Clustering of all cells, showing ten subsets from the two samples. **C** The marker genes for each subset are listed. **D** The subcluster of T cells, annotations, marker genes and cell ratios of each subset. **E** The flow cytometry results of γδT cells in different groups. **F** The statistics of ratios of γδT cells in all live cells for different groups. **G** The GO enrichment terms in T cells of TLR9agonist_LW and WT_LW. **H** The healing process of WT_LW, TCRδ−/−_LW and TLR9agonist activated WT and TCRδ−/− LW. *n* = 3–5 for each group. All error bars mean ± SD.

The activation of TLR9 has been reported to induce the inflammation and polarization of T helper 1cell (Th1), Th17/Th23, promotion of release of type I interferon (IFN), Interleukin-6 (IL-6), IL-1β so forth [20, 26]. In order to investigate the specific characteristics influenced by the activation of TLR9 in wounds, we employed the single-cell RNA sequencing (scRNA-seq) to visually compare immune cells and inflammatory factors and cytokines under the influence of TLR9.Tissue samples were collected from the center of the wound (φ = 5 mm) on the second day after scab detachment (SD2), comprising two groups: WT_LW and TLR9agonis_LW, each consisting of six mice. These samples were subsequently subjected to analysis using the 10x scRNA-seq platform (Fig. 3A). Following cell filtering, unsupervised clustering utilizing Seurat software assigned cells into distinct clusters based on their global gene expression patterns. This was followed by the categorization of these clusters into primary cell classes at the first level. Ten cell types were defined: T cells (TC), fibroblasts (Fib), myeloid cells (Myl), keratinocytes (Ker), pericyte cells (Perc), neural crest-derived cells (Neur), endothelial cells (Endo), other cells (Others), neutrophils (Neu), lymphatic endothelial cell (Lyen) (Fig. 3B). The composition of each main cluster was listed so that the proportion of cells from two groups could be identified across all cell clusters. Marker genes for each main cluster were shown in the heatmap and listed in Fig. 3C. Subsequently, we performed enrichment analysis of overall gene expression in both groups. GO functional enrichment revealed significant up-regulation of many entries related to hair follicle development、epithelial tube morphogenesis, gland and appendage morphogenesis in the TLR9agonist_LW group, as well as hair follicle stem cell-related genes such as Krt17 [27], Sox9 [28], Msx2 [29], Lgr5 [30] (Fig. S3B, C). In contrast, the WT_LW group mainly upregulated genes associated with myeloid cell differentiation, phagocytosis, WNT and TGFβ (Fig. S3C).

To better analyze the mechanisms underlying the effects of TLR9 activation on wound healing and HF regeneration, we first annotated the two most important stromal cell populations, keratinocytes and fibroblasts, based on previously reported markers. Keratinocytes were divided into permanent epidermis (EPI), upper and middle hair follicles and cells defined as or highly related with hair follicle stem cells (HFSC) (Fig. S3D). The subclusters of keratinocytes were further divided and annotated according to markers reported before [11, 31–33]. The GO enrichment analysis also showed genes related with hair follicle morphogenesis/hair cycle and skin development were upregulated in TLR9agonist_LW, while genes related with extracellular matrix organization and stem cell differentiation enriched in WT_LW (Fig. S3E). Furthermore, in order to clarify the effects on keratinocytes and the resulting changes more clearly, we conducted detailed analysis of the development trajectory of keratinocytes and their interaction with immune cells, which can be seen in Figs. 5 and 6.

Similarly, we specifically focused on the main clusters that were identified as fibroblasts and subjected them to a secondary round of clustering (Fig. S3F, G). Fibroblasts play a crucial role as the primary mesenchymal cells in the dermal layer of the skin, and different subclusters of fibroblasts are spatially distinct with significant functional diversity. Generally, dermal fibroblasts originate from several distinct lineages: (1) the upper lineage consists of papillary fibroblasts (PF), which are in direct contact with the epidermis and contribute to the dermal component of hair follicles; (2) the lower lineage comprises reticular fibroblasts (RF), responsible for synthesizing most of the extracellular matrix (ECM) proteins, and lipo-fibroblasts (LF), which give rise to preadipocyte progenitors in the hypodermis.

Additionally, certain populations of fibroblasts play unique roles in WIHN and are therefore of particular interest to us, namely dermal papilla (DP) and dermal condensate (DC), which possess unique transcriptional characteristics along with the general fibroblast population [34]. DP is located at the base of mature hair follicles and serves as the principal signaling niche regulating hair follicle activities [9, 35]. Dermal condensate (DC), originating from the papillary fibroblasts, is believed to be the progenitor of DP during embryonic development. In our dataset, we defined six types of fibroblasts consisting of ten subclusters based on previously established marker genes: Inhba+Prdm1+Ereg+ Fabp5+Sdc1+ representing papillary fibroblasts (PF), Pappa2+Mdk+Wnt5a+ Hhip+Vcan+ indicating dermal papilla (DP), Fst+Twist2+Smad3+ characterized dermal condensate (DC), Plac8+Gpx3+Mest+ indicating reticular fibroblasts (RF), Plin2+Ptprc+Lyz2+ indicating myeloid-derived adipocyte progenitors (Macf), and Birc5+Mki67+Tagln+ representing myofibroblasts (MF) (Fig. S3F, G).

In the WT_LW group, the initial phase of dermal repair was mediated by the lower lineage fibroblasts, particularly the reticular fibroblasts, which were associated with the organization of extracellular matrix organization, external encapsulating structure organization, as revealed by gene ontology enrichment analysis (Fig. S3G). In addition, the fibroblasts in WT_LW highly enriched terms of transforming growth factor beta (TGF-β) receptor signaling pathway, which was known associated with fibrosis [36]. In contrast, the TLR9agonist_LW wounds exhibited a higher proportion of upper lineage Crabp1+Prdm1+ papillary fibroblasts, which are known to have the capacity to support hair follicle initiation [37, 38]. Interestingly, we also identified the presence of Fst+ Twist2+Smad3+ dermal condensate (DC) cells in this dataset (Fig. S3F). During embryonic hair follicle development, Dc acts as a signaling niche that promotes epithelial placode growth and subsequently contributes to hair follicle morphogenesis [35]. In GO enrichment analysis, we observed the genes related with morphogenesis of embryonic epithelium, regulation of tumor necrosis factor (TNF) production were highly regulated in TLR9agonist_LW (Fig. S3G), which reminded us of the positive correlation between TNF-α [39] or IL-1β [13] with hair growth in wounds. Additionally, as observed by Gay et al. [8], the WNT signaling pathway was also more significant in low-regeneration group at similar late wound healing, after the end of re-epithelialization. Given the significantly increased proportion of specific cell populations and enrichment terms in the TLR9agonist_LW group during the critical period of hair follicle regeneration, it is plausible to suggest that they may provide an adequate mesenchymal component for subsequent hair follicle formation in the TLR9-activated group.

TLRs are reported to be expressed in antigen-presenting cells, establishing a crucial link between pathogen recognition and the activation of both innate immune effector mechanisms that restrict pathogen replication and adaptive immunity initiation [17]. To further explore the impact of TLRs on the host’s overall immune response, we conducted analyses of various immune cell types using single-cell RNA sequencing.

Firstly, neutrophils, as the primary responders and critical mediators of the innate immune system, play a vital role in the recruitment and differentiation of monocytes [40], particularly during the early stages of immune response. In our scRNA sequencing data obtained from the late stage of wound healing, we observed that most subclusters of neutrophils exhibited comparable proportions in both experimental groups (Fig. S4A). Macrophage-monocytes and dendritic cells are the main cells expressing TLRs and equipped to coordinate the activation of other immune cells as well as tissue repair [20]. Five types of macrophage-dendritic cells were identified, according to markers reported in literature [41]: Inhba+Ptgs2+ Mmp12+pro-inflammatory macrophages (PIM), Ccl8+Mrc1+ Folr2+Fcgr1+ anti-inflammatory macrophages (AIM), Irf7+Il3ra+ plasmacytoid dendritic cells (pDC), Cd74+Cd86+Cd207+Rgs1+ monocyte-derived dendritic cell1 (mDC), Cd8a + Cadm1+ Cadm3+ Clec10a+ Cpvl + conventional dendritic cell (cDC) (Fig. S4C). As speculated, TLR9 agonist, as a type of pro-inflammatory agent, increased the presence of PIM in wounds while decreasing AIM (Fig. S4C). Functional enrichment analysis of the two groups revealed that the TLR9 agonist upregulated numerous items related to protein synthesis and secretion in the large wound group, in consistent with the macrophage activation response to TLRs as reported by Fitzgerald et al. [17]. Further subcluster comparisons and enrichment analysis of macrophage-dendritic cells demonstrated that macrophage-dendritic cells were stimulated and activated in the TLR9agonist group (Fig. S4C). pDC, as one of the cell types expressing TLR9 and recognizing self-nucleic acids, have been shown to quickly migrate into wound and produce proinflammatory cytokines such as type I interferon (IFN). However, we did not observe an increase of number of pDC in the late-stage wound of TLR9agonist_LW. We suppose this attributed to rapid recovery of pDC number [20]. However, in the TLR agonist group, pDC showed significant upregulation of pathways such as toll-like receptor signaling pathway and cytosolic DNA-sensing pathway, as well as activation-related pathways like NF-kb and MAPK (Fig. S4D).

### γδT cell numbers significantly increased, which was further confirmed to be correlated with the activation of TLR9

The activation of TLRs in various cells can also lead to the initiation of adaptive immunity [17]. In our single-cell data, T cells were found to be most abundant immune cells, inspiring our interest of the function and changes in T cells (Fig. 3B). The subclustering of T cells resulted in five main subsets including CD4-IL17a+Cd7+ γδT cell (γδT), CD4+Ifng+Il18+ T helper 1 (Th1), CD4+Gata3+Il4+ T helper 2 (Th2), Foxp3+Ctla4 + T regulatory cell (Treg), Cd8b1+Nkg7+ cytotoxic T cell (CTL), based on markers from published research [42] (Fig. 3D). After defining cell subsets, we observed prominent increase in the number and proportion of γδT cells (Fig. 3D) and numerous enriched GO terms related with activation and differentiation of γδT cells in the TLR9 agonist_LW group (Fig. 3G). The increased γδT cells were primarily IL17-producing γδT cells from the dermis, characteristic of Cd27-Cd44+ IL-17a+Il7r+ Blk+ Maf+ Rorc + [43]. The changes in γδT cells were further confirmed by flow cytometry (Fc) of wounds with different levels of activation of TLR9 (Fig. 3E, F). The wounds with enhanced tissue damage (WT_eLW) also resulted in an increase in the number of γδT cells, further suggesting that γδT cells are influenced by the degree of tissue damage and the activation of TLR9, while the number of γδT cells decreased in TLR9 knockout mice (Fig. 3E, F). These results may indicate that the activation of TLR9 may have great influence on γδT cells.

Given the close association between γδT cells and TLR9, we hypothesized that γδT cells are key cells mediating the effects of TLR9 activation on wound repair and regeneration. Therefore, we utilized the TCRδ−/− mice which lack γδT cells. We found that mice with TCRδ−/− mice exhibited significantly slower wound healing rates (Figs. 3H and 5I). Moreover, in TCRδ−/− mice, the promotion of wound healing by TLR9 agonist was abolished upon injection (Fig. 3H). Furthermore, we also found that the impact of TLR9 agonists on WIHN also relies on γδT cells (Fig. 5H, I). These results collectively indicate that γδT cells are critical cells mediating the actions of TLR9.

### The CCL2-CCR2 axis, known to promote the recruitment of γδT cells to the periphery, was found to be influenced by TLR9, accounted for the increase of γδT cells

**Fig. 4:**
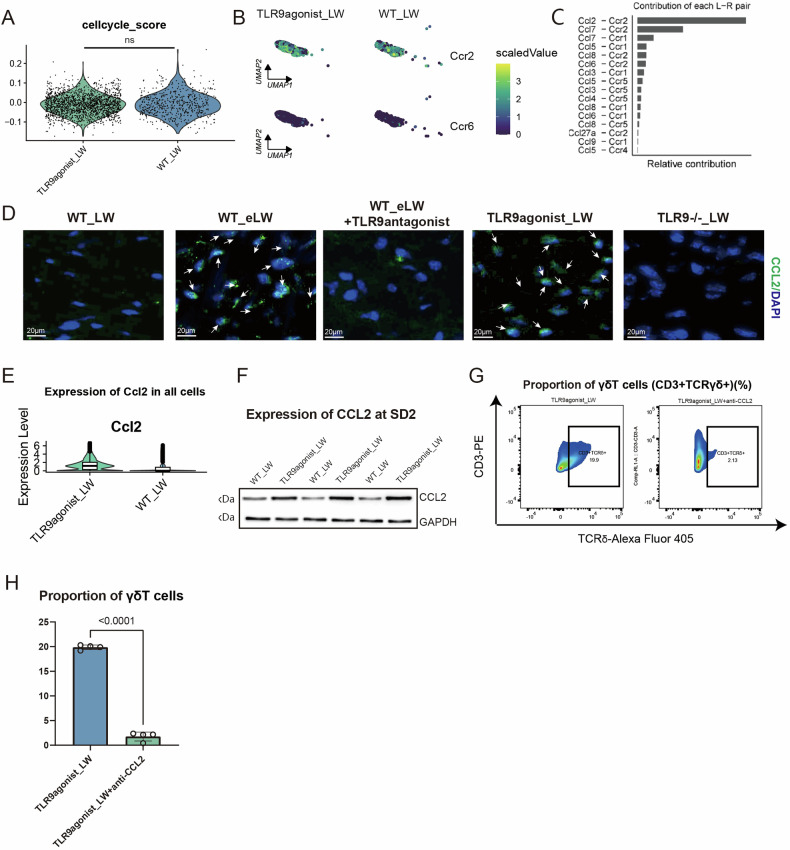
The increase of migration of γδT cells, which could be accounted by the enhancement of CCL2-CCR2 interaction. **A** The score of cycle cycle-related genes in γδT cells. **B** The expression of Ccr2 and Ccr6 in γδT cells of two groups. **C** The L-R pair contribution of CCL family. **D** Immunofluorescence staining was performed to visualize the expression of the CCL2 protein in the dermal part of skin wounds with different treatments at SD2. Scale bar: 20 μm. **E** The expression of Ccl2 in all cells from TLR9agonist_LW and WT_LW. **F** The expression of CCL2 in wounds at SD2 was detected by western blotting. GAPDH was used as loading control. **G**, **H** The proportions of γδT cells in wounds treated with TLR9agonist +anti-CCL2 antibody (26161-1-AP, Proteintech) (10 μg per time for per mouse) at PWD 0,3,6 or TLR9agonist +control-IgG from same species. *n* = 4 for each group. All error bars mean ± SD.

To explore the source of the increased γδT cell numbers, we speculate that TLR9 may promote the increase in the number of γδT cells by enhancing their local proliferation. We scored the proliferation-related genes of γδT cells with AddModuleScore in Seurat and found that the cell cycle-related genes of γδT cells in the TLR9 stimulated group didn’t increase, indicating the increase of γδT cells in wound might not be caused by enhancement of local expansion or proliferation (Fig. 4F).

Due to the constant migration and movement of γδT cells between lymph nodes and peripheral tissues, their migration speed and pattern undergo changes in cases of tissue infection or injury [44]. We therefore investigated whether the increase in γδT cell numbers result from an increase in their migration into the skin. As reported by McKenzie et al., in steady-state conditions, CCR6 controls γδT 17 trafficking to the dermis; however, in cases of tissue damage, CCR2 controls the rapid migration of γδT 17 to damaged sites [45]. Therefore, we first observed and compared the expression of CCR2 and CCR6 in γδT cells. We found that almost all γδT cells in TLR9-activated wounds were CCR2^hi^ CCR6^lo^ cells (Fig. 4B). The expression of CCR2 in γδT cells was significantly increased in TLR9 agonist wounds (Fig. 4B). To delve deeper into the ligands of CCR2, we isolated γδT cells and analyzed their interactions with other cells using cellchat (Fig. 4C). Notably, the ranking of the contribution of the CCL family L-R pair in cellchat affirmed the pivotal role of CCR2 (Fig. 4C). Among the various CCR2 ligands, CCL2 emerged as the most potent and prominently expressed, with significant upregulation observed in the TLR9 agonist group. Immunofluorescence staining and western blot analyses further underscored the strong association between CCL2 expression and TLR9 activation levels, peaking in the TLR9 agonist or enhanced injury groups and diminishing in TLR9 knockout mice (Fig. 4D–F). Consequently, we postulated that TLR9 may facilitate the chemotaxis of γδT cells via the CCL2-CCR2 axis. Intriguingly, concurrent administration of anti-CCL2 or Ctrl-IgG with TLR9 agonists revealed that the reduction in γδT cell numbers at the wound site mediated by anti-CCL2 antibodies counteracted the effects of TLR9 agonists on increase of γδT cell (Fig. 4G, H).

### The role of γδT in promoting hair follicle regeneration is achieved through Areg rather than IL-17

**Fig. 5:**
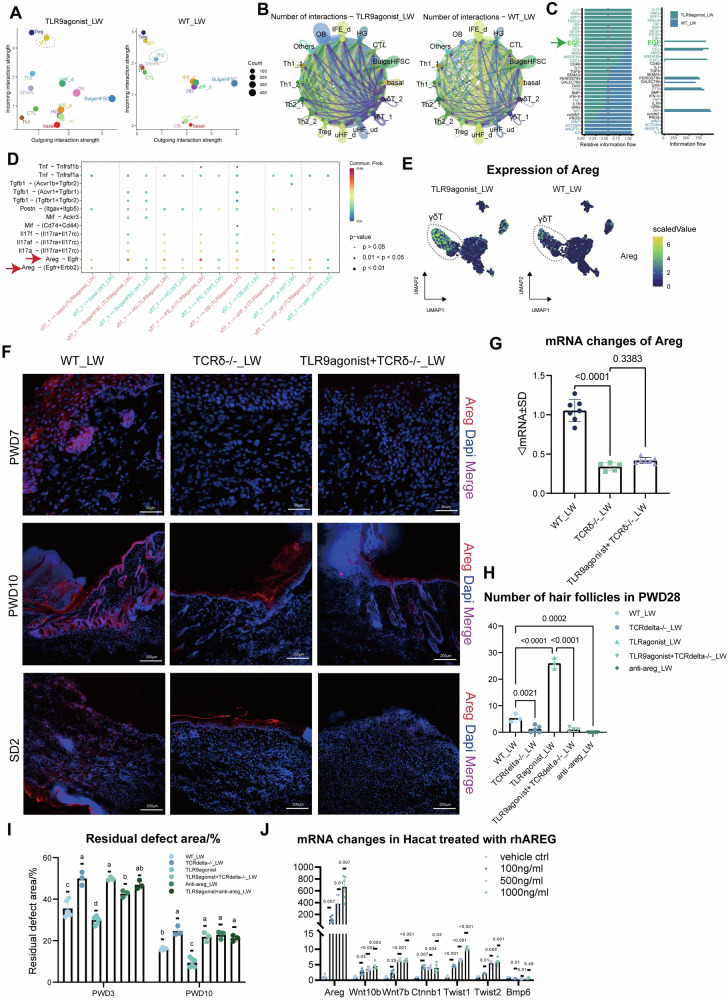
The Areg mediates the interaction between γδT cells and keratinocytes. **A** The information stream plot indicating the incoming and outgoing interaction strength in T cells and keratinocytes. The most intense outgoing interaction of T cells to keratinocytes in TLR9agonist_LW was launched by γδT cells, but in WT_LW it was Th2. **B** The differential interaction pathways in TLR9agonist_LW and WT_LW. **C** The interaction numbers between T cells and keratinocytes in TLR9agonist_LW and WT_LW. **D** The bubble diagram showing the pathways mediating the interaction between γδT cells and keratinocytes in two groups. **E** The expression level of Areg in γδT cells. **F** Immunofluorescence staining was performed to visualize the expression of the AREG protein in skin wounds with different treatments at PWD7, PWD10, SD2, respectively. **G** The mRNA changes of areg in WT_LW, TCRdelta−/−_LW and TLR9agonist+ TCRdelta−/−_LW at SD2. **H** The number of hair follicles at PWD28 in different group. **I** The healing speed differences in different group. The injection of anti-AREG (15 μg per time per mouse. HY-P77868, MedChemExpress) or Control-IgG (30000-0-AP, Proteintech) into the wound bed was conducted at PWD 0,3,6,10. **J** The mRNA changes of *Areg, Wnt10b, Ctnnb1, Twist1, Twist2* in Hacat cells after culturing with various concentrations of rhAREG for 48 h.

Although anti-CCL2 reduced the number of γδT17 cells and had a significant inhibitory effect on wound healing and hair follicle regeneration, we were still unsure which molecule was responsible for promoting hair follicle regeneration via γδT cells. It has been previously reported that the γδT17 promotes WIHN by secretion of fgf9 and then activates the WNT pathway of keratinocytes [46]. However, in our scRNA data, the levels of Fgf9 mRNA are extremely low in γδT cells both in TLR9agonist_LW and WT_LW (Fig. S5B). Therefore, we hope to investigate whether there is another molecule secreted by γδT cells that exerts significant influence on the WIHN level. We analyzed the cell interactions between T cells and keratinocytes using cellchat (Figs. 5A, S5A), observing the increased number of T-ker interactions in the TLR9 agonist group, and prominent importance of γδT cells in the interaction with keratinocytes(Fig. 5A, B). Among the increased pathways, IL-17 was the most prominent (Fig. S5C, D). Through Cellchat and featureplot, we identified that IL-17 was produced by γδT17 cells and mainly received by upper hair follicle cells, interfollicular keratinocytes and hair germs which may be closely related to hair follicle development (Fig. S5D). The increased of IL-17A was also confirmed in both scRNA data and WB staining (Fig. S5B, E). Given the reported role of IL-17A in promoting wound healing [47] and the stemness of keratinocytes [48, 49], we hypothesized that the action of γδT17 cells is also achieved through IL-17A. However, by injecting anti-IL-17A into the wound site early in the experimental period, we found that although IL-17A had a significant effect on wound healing speed, antagonizing IL-17A did not affect the hair follicle regeneration promoted by TLR9 agonists (Fig. S5F–H). Moreover, according to literature, in WIHN model without intervention, there was no difference in regeneration outcomes between IL-17a/f−/− mice and WT mice [13]. Therefore, we speculated that the action of γδT cells is achieved through other signaling molecules.

The EGF family was also a highly represented signaling molecule in T cell and keratinocyte interactions, and one of the significantly different molecules between the two groups (Fig. 5C, S5I, J). Akiyama et al. found Bulge HFSC was highly co-localized with EGFR during embryonic development [50], and EGFR KO mice exhibit impaired hair follicle differentiation and multiple hair shaft abnormalities, indicating the crucial role of EGF signaling in hair follicle development. Through cellchat L-R scoring, we found that Areg was the strongest acting signal in the EGF family (Fig. 5D). The areg signal was involved in almost all γδT17-related actions on various keratinocytes, with upper hair follicle cells being the most significant cell populations involved in hair follicle development (Fig. 5D). The increased expression of Areg was also verified in scRNA data, immunofluorescent staining and RT-qPCR analysis (Fig. 5E–G). Mice without mature γδT cells has significantly decreased expression of Areg, and adding TLR9agonist into wounds couldn’t rescue the low expression of Areg (Fig. 5F, G). These results help proved the correlation between TLR9 activation, γδT cells and expression of Areg.

As a member of the EGF family, AREG is considered one of the low-affinity ligands of EGFR and has been implicated in a variety of physiologic processes, including regulation of keratinocyte proliferation and gland development [51]. While AREG has been shown to influence wound healing speed [52], its impact on follicle development and whether it affects the de novo formation of follicles in adult individuals in the context of WIHN remains unclear. Therefore, we aimed to investigate the hypothesis that AREG may influence WIHN levels and that the function of γδT cells is primarily achieved through AREG. By injecting monoclonal antibodies against AREG to antagonize its function, we observed a significant reduction in wound healing and hair follicle regeneration, highlighting the role of AREG (Fig. 5H, I). Additionally, to elucidate the mechanism by which AREG acts on keratinocytes, we conducted an in vitro study using HaCaT cells. Various concentrations of recombinant AREG (rh-AREG) were added to the cell cultures, and through RT-qPCR analysis, we observed a significant promotion of cell proliferation (Fig. S6A) and upregulation of genes associated with hair follicle regeneration, such as *Wnt10b, Ctnnb1, Twist1, and Twist2*, while lower expression of *Bmp6*, which is known to be adverse for WIHN (Fig. 5F). Concurrently, we also observed a significant increase in the expression level of *Areg* itself upon addition of recombinant AREG, suggesting the presence of a positive feedback loop (Fig. 5F), which was consistent with the results in cellchat where keratinocytes are recognized as key recipients and responders to AREG (Fig. S5I, J). Therefore, we hypothesized that AREG produced and secreted by γδT cells may act on keratinocytes via paracrine signaling, boosting the production of AREG by keratinocytes themselves, and leading to a localized rapid elevation of AREG levels and enhancement of its effects. To obtain further information regarding the association of AREG with healing, particularly WIHN, we conducted additional analysis of biological samples from different WIHN outcomes sourced from public databases (GSE159939). We discovered that AREG exhibits relatively high expression in the high-WIHN outcome area (center of wounds in Mus musculus mice, or global area in Acomys spiny mice), while showing relatively low expression in the peripheral wounds of Mus musculus mice, which characteristic as low-WIHN outcome mice (Fig. S6B). We believe this further support the potential positive role of AREG in WIHN.

### AREG was found to be associated with development of keratinocytes

**Fig. 6:**
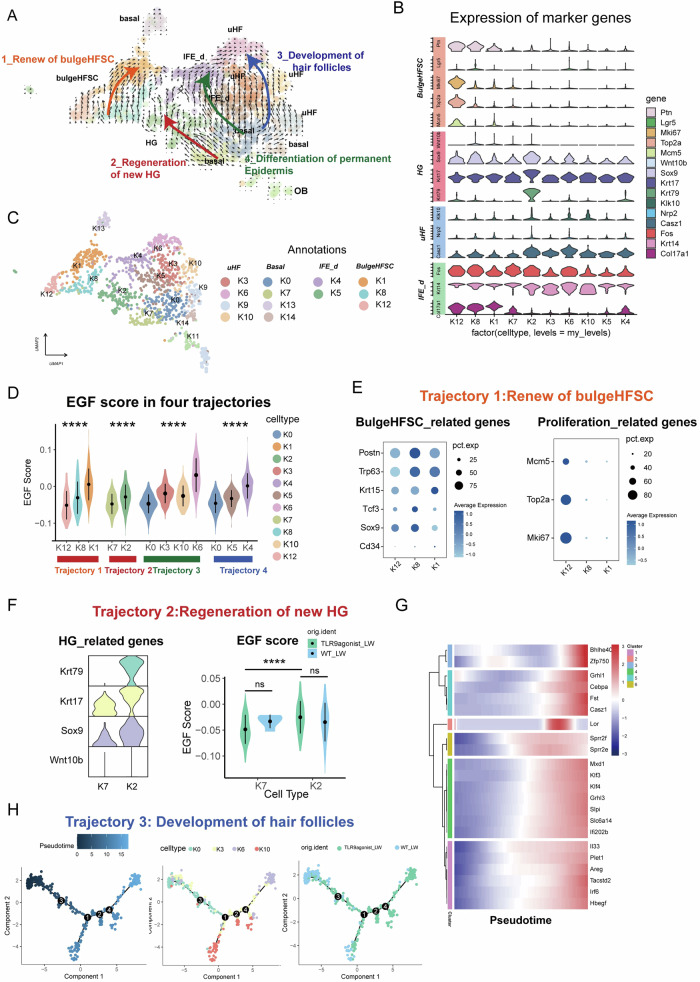
The development trajectories of keratinocytes and the involvement of AREG. **A** The RNA velocity analysis of keratinocytes. Trajectory 1: self-renewal of bulgeHFSCs; trajectory 2: regeneration of new hair germs (HG); trajectory 3: development of hair follicles; trajectory 4: differentiation of the permanent epidermis. **B** The annotation of subsets in keratinocytes. **C** The expression of marker genes used for identification of development trajectories. **D** The scores of EGF in every trajectory. **E** The marker genes of bulgeHFSC and proliferation related genes. **F** The HG related genes and EGF scores changes in trajectory 2. **G** The heatmap plotting differential gene expression from pseudotime analysis in trajectory 3.**H** The pseudotime analysis of main cells in trajectory 3. Vlnplot and box plot graphs indicated the value of minimum, first, quartile, median, third quartile, and maximum. *p < 0.05, **p < 0.01, ***p < 0.001, and ****p < 0.0001.

To elucidate the changes in behavior and differentiation tendency of keratinocytes under TLR9 agonist stimulation, we analyzed the development and differentiation trajectory of keratinocytes in two groups using monocle2 and RNA velocity. Unsupervised clustering of RNA velocity in keratinocytes revealed four major differentiation pathways. Based on cell annotation and spatial localization of annotated cells (Fig. 6B), we determined four developmental trajectories, namely as: self-renewal of bulgeHFSCs (trajectory 1), regeneration of new hair germs (HG) (trajectory 2), development of hair follicles (trajectory 3), and differentiation of the permanent epidermis (trajectory 4) (Fig. 6A).

Trajectory 1 originated from the highly proliferative Cluster 12 cells characterized by Mki67+ Ccnb1+ Pcna+ and terminated at Lhx2+Cd34+ Ptn + Lgr5+ bulgeHFSC (K8, K1), suggesting that pathway one may represent the proliferation and self-renewal of bulge HFSCs (Fig. 6E). Trajectory 2 was initiated by IFE (K7) which did not express any HG markers and culminated in K2 characterized by highly expressed HG markers (Krt79+Krt17+Sox9+) (Fig. 6F) [10], which was mainly derived from the development of IFE with lineage plasticity and migration after injury in WIHN, indicating that trajectory 2 represented the de nono formation of new HGs in WIHN. Trajectory 3 originated from moderately differentiated keratinocytes and gradually underwent differentiation and maturity towards hair follicle, indicating that trajectory 3 represented the process of hair follicle development (Fig. 6A). Trajectory 4 originated from basal-like keratinocytes and underwent gradual differentiation, indicating that trajectory 4 represented the process of differentiation of the interfollicular epidermis.

To elucidate the distinct cellular states during epidermal differentiation and the generation of new hair follicles in the context of WIHN, we initially extracted the major cell clusters (clusters K0, K3, K6, K10) mainly composed of trajectory 3 and investigated the differentiation state of these keratinocytes through pseudotime analysis (Fig. 6H). As pseudotime progressed (Fig. 6H), the expression of many genes proved to be involved in maturity and differentiation in hair follicles such as Bhlhe40, Zfp750, Grhl1, Cebpa et al. [32] increased, indicating the developmental pattern (Fig. 6H). Concurrently, there was a higher concentration of TLR9agonist cells in the late stages of the pathway, suggesting a stronger tendency towards hair follicle differentiation (Fig. 6H). To understand the role of AREG in hair follicle development, we also performed EGF scoring. During the process of hair follicle development, both the expression of AREG and EGF scoring increased as the developmental pathway advanced (Fig. 6D, H), revealing the involvement of Areg and EGF in this trajectory. These results suggest that the expression of Areg and EGF were highly synchronous with multiple development trajectories of keratinocytes.

## Discussion

Since the discovery of the WIHN phenomenon, one of the most interesting and urgent phenomena to study is the requirement of sufficiently large wound areas for hair follicle regeneration. These hypotheses encompass a range of factors, including the unique mechanical topology of large wounds, the extent of tissue damage within such wounds, and the varying concentration and spatial distribution of signaling molecules that either promote or hinder regeneration in large wounds [53]. These hypotheses have received partial support through empirical evidence. Interestingly, previous investigations have documented a positive correlation between the size of wounds and their regenerative potential, whereby larger or more severely damaged wounds elicit more robust regeneration [4, 15]. In this study, we harnessed diverse wound types that encompassed different levels of injury on the dorsal skin to search for early, vital events linked damage-associated signals and the launch of repair and regeneration.

The recognition of damage-associated molecular patterns (DAMPs) and pathogen-associated molecular patterns (PAMPs) by pattern recognition receptors (PRRs) represents a fundamental mechanism in the initial response to tissue damage or pathogen invasion. In the aftermath of the activation of DAMPs and PAMPs, two pivotal steps must be orchestrated to facilitate tissue repair and regeneration: (1) the coordinated mobilization and activation of pertinent precursor cells, facilitating the reconstitution of damaged tissue structures, and (2) the induction of morphogenetic and regenerative pathways within both precursor cells and stromal cells [54]. The elucidation of the impact of PPR activation on the immune microenvironment may shed light on this inquiry. Activation of PPRs elicits intricate inflammatory responses, characterized by the recruitment, proliferation, and activation of diverse hematopoietic and non-hematopoietic cells, thereby initiating and coordinating tissue repair and defense responses [55].

In this study, we firstly identified the influence of DAMPs release and recognition of PPRs on the immune microenvironment and the repair process by assessing the number of regenerated hair follicles associated with varying degrees of injury. By analyzing bulk RNA sequencing data from large wounds with different levels of damage, we observed variations in the activation levels of TLRs and specifically TLR9. Previous research has demonstrated the involvement of TLRs, a significant subtype of PPRs, in damage sensing and the initiation of tissue repair and regeneration [56]. TLRs have also been implicated in the regeneration of skin wounds. For instance, TLR3 activation triggered by the detection of released dsRNA from injured cells upregulates IL-6 expression and induces STAT3 phosphorylation in epidermal keratinocytes and finally promotes the regeneration of hair follicles [9, 15]. However, TLR9 has often been overlooked due to its lower expression levels, being presumed to be expressed solely in response to skin injury or external stimuli in human and mouse skin [57, 58]. Gregorio et al. [20] found that tissue damage can activate plasmacytoid dendritic cells (pDCs) through TLR7 and TLR9-dependent pathways to enhance skin wound healing, without addressing the effect of TLR9 activation on various types of immune cells and whether they can affect hair follicle regeneration. Moreover, our study has not only confirmed the involvement of TLR9 in wound healing but also, for the first time, revealed its role in reactivating embryonic-like developmental programs by modulating the late immune microenvironment of the wound.

Furthermore, we discovered that the enhancement of large wounds compared to ordinary large wounds is primarily facilitated by the release of free mtDNA, which activates TLR9. During cellular trauma, mitochondrial DAMPs, including mtDNA containing CpG DNA repeats, have been observed to be released and activate TLR9, GMPAMP synthase-stimulator of interferon genes (cGAS-STING), and neutrophil extracellular traps (NETs) [24]. Previous studies have frequently associated mtDNA with NETs, which was demonstrated to impede WIHN, thus implying a negative effect of mtDNA on hair follicle regeneration [59]. Through meticulous in vitro and in vivo investigations, we discovered that lower levels of mtDNA can stimulate TLR9 activation and hair follicle regeneration (Fig. 2G). Although the WIHN phenomenon is absent in human skin, mtDNA has also been detected in cases of human tissue damage, accompanied by increased TLR9 expression, demonstrating a similar mechanism [24]. These findings offer novel insights into the activation of signaling receptors implicated in tissue damage, the orchestration of immune responses, and ultimately, the outcome of tissue repair and regeneration. Therefore, we proposed the hypothesis that greater tissue damage and promotes WIHN by inducing heightened activation of TLR9 pathway through mtDNA release in a dose-dependent manner.

As one of the key barrier cells residing in the skin, gamma delta T cells (γδT) have been found to play a vital role in detecting skin integrity, maintaining skin homeostasis, aiding in wound repair, preventing infection, and preventing malignant tumors [44, 60]. Specifically, wounding has been shown to up-regulate epidermal-derived IL-1α, which serves as a potent activator of γδT cells. In addition, γδT cells have also been suggested to be associated with Toll-like receptors (TLRs). The number of γδT cells decreases in skin wounds of TLR3−/− mice [15]. In addition, recent evidence has emerged that γδT plays a role in cutaneous wound healing [61]. In our study, we also observed that under stronger TLR9 activation levels, either through administering TLR9 agonists or enhancing tissue damage, there was an increase in the number of γδT cells in the wounds. Mice lacking γδT cells exhibited significantly impaired healing speed and follicle regeneration, and they could’t be rescued by TLR9 agonists or enhanced tissue damage, indicating that the function of TLR9 was mediated through γδT cells. Consequently, our research further underscores the critical role of γδT cells in sensing signals related to tissue damage and their key contribution to tissue repair and regeneration processes.

Previous literature has proposed several findings regarding the mechanisms by which γδT cells exert their effects on healing outcomes and their impact on keratinocytes. Activated γδT cells promote hair follicle stem cell (HFSC) proliferation and migration, thereby facilitating injury-induced hair regeneration [46, 47]. Furthermore, Gay [46] et al. proved that γδT plays a vital role in WIHN by secreting FGF9. However, in the context of larger wounds than models studied by Gay et al. [46], the FGF9 was barely detected in both groups from our data, suggesting that γδT cells may affect regeneration outcomes through other pathways in this condition. Additionally, our findings demonstrate that although most of the recruited γδT cells were IL-17-producing γδT cells, the addition or antagonism of IL-17A did not alter hair follicle regeneration outcomes. By analyzing the intercellular communication between γδT and multiple keratinocytes, we found γδT cells mainly affected keratinocytes by secreting AREG. The production of γδT promotes AREG secreted by keratinocytes through paracrine signaling, leading to a positive feedback loop that rapidly and significantly increases AREG levels.

AREG has been demonstrated to function as one of the alarm systems following injury. Recent studies have highlighted its crucial role in promoting epithelial cell proliferation and differentiation, particularly in the late stages of wound healing where it aids in resolving inflammation [62]. However, whether this promotion of keratinocyte differentiation is beneficial for WIHN remains unclear. Some observations suggest that AREG promotes interfollicular epidermal fate at the expense of consuming keratinocytes’ fate [63]; yet, other research has found that AREG enhances hair follicle neogenesis [64]. Given the impact of the late-stage inflammatory environment on cell fate and the complexity of stem cell fate in WIHN, we think that further experimental evidence is needed to fully understand the role of AREG. Our single-cell analysis and experiments revealed that AREG affects the expression of genes such as *Twist1, Twist2, Wnt10b*, and *Ctnnb1* in keratinocytes and significantly influences the multiple processes and cell fates of keratinocytes, including the process of hair follicle development, the *de nono* formation of hair germs and the self-renew of bulge HFSC. Our study not only sheds new light on the role of γδT cells in wound-induced hair neogenesis (WIHN), but also provides explanations for how these cells interact with injury-related signals and ultimately affect the behavior of matrix cells involved in repair.

Our work raises more questions that need to be addressed for the field. Firstly, one of the critical questions is whether similar mechanisms exist in humans. As previously discussed, TLR9 expression is up-regulated in human keratinocytes after injury [57], and γδT cells also exist in the human dermis. Furthermore, AREG-producing γδT cells have been identified in tumors, promoting the proliferation of tumor epithelial cells and exhibiting wound healing functions [65]. Therefore, it remains to be determined whether γδT cells in human skin will be similarly affected by TLR9 stimulation and produce AREG in response to skin injury. Additionally, aside from dermal γδT cells, will skin injury and TLR9 activation also affect epidermal DETCs or macrophages? DETCs, as one of the critical cells that reside in the epidermis and monitor the status of adjacent keratinocytes through dendritic projections, undergo rapid morphological and functional changes in response to epithelial damage [66]. Although we were unable to collect sufficient DETCs for analysis due to the limit of single-cell transcriptome cell numbers in this study, the extent of tissue damage and how TLR9 affects DETCs remain important issues worthy of further research. In apart, lower WNT signaling in macrophages and fibroblasts and phagocytosis of macrophages were observed in TLR9 activated group within a few days after re-epithelialization, as reported in Gay et al. [8]. Could TLR9 function through similar mechanisms that inhibit late excessive and prolonged WNT activation? In the future, we will explore the detailed mechanisms by which TLRs influence other processes of skin repair and regeneration and to investigate whether any of these mechanisms could explain the healing outcome changes and the lack of WIHN in humans.

## Materials and methods

### Ethical approval

The experimental procedures undertaken in this study were granted ethical approval by the Institution Review Board of West China Hospital of Stomatology, Sichuan university (Approval No. WCHSIRB-D-2023-018).

### Sex as a biological variable

Our study examined male mice because male animals exhibited less variability in phenotype.

### Excisional wound model and implantation procedures

Male wildtype C57BL/6J mice (Dossy Experimental Animals Co., Ltd.) and C57BL/6Smoc-Tlr9em1Smoc mice (TLR9^−/−^ mice) (Cat. NO. NM-KO-190168) ((Shanghai Model Organisms Center Inc., Shanghai, China, aged 6–8 weeks and weighing approximately 20 g, were utilized for the research. Genotyping of the Tlr9 locus was performed using the following primers: 5′-GCTCTCCCACTTTCTCTTCCTCTC-3′ and 5′-TGCCGCCCAGTTTGTCAG-3′ for the wild-type allele, and 5′-TGCCGCCCAGTTTGTCAG-3′ and 5′-ACGGGAAAAGGGTGGGTGTG-3′ for the Tlr9 knockout allele. The mice were housed under standard conditions including a temperature range of 21–27 °C, a humidity level of 40–70%, and a 12-h light–dark cycle with ad libitum access to food. The number of animals utilized for each experiment is specified in the figure legends. Circular full-thickness wounds with diameters of 0.6 cm or 1.8 cm were created on the dorsal skin of the mice. The mice were divided into several groups for further study: large wounds (1.8 cm diameter) treated with PBS (WT_LW) or TLR9 agonist in the wound bed (TLR9agonist_LW), and small wounds (0.6 cm diameter) treated with PBS (WT_SW). Additionally, to investigate the effects of different degrees of damage, three types of large wounds (1.8 cm diameter) with additional incisions were designed and used: dotted WT_eLW with 6–8 poke points through the epidermis and dermis, parallel WT_eLW with 6-8 short incisions parallel to the cutting edge, and vertical WT_eLW with 6–8 radial short incisions perpendicular to the cutting edge. Mice were euthanized at 1–4 weeks after the surgery, and round full-thickness samples with diameters of 10 mm (for small wounds) or 25 mm (for large wounds) were harvested. To investigate the function of CCL2, IL-17A, and AREG, anti-CCL2 (26161-1-AP, Proteintech) at a dosage of 10 μg per mouse, or anti-IL-17A (PAB30184, Bioswamp) at a dosage of 10 μg per mouse, or anti-AREG (66433-1-Ig, protech) at a dosage of 15 μg per mouse was injected into the wound bed at postoperative day (PWD 0, 3, and 6. Rabbit IgG served as the control antibody (30000-0-AP, Proteintech) in all cases.

### RNA isolation and real-time PCR

For in vivo mouse samples, isolation of total RNA was conducted after homogenization using the RNeasy Mini Kit (Qiagen, 74106) as appropriate. RNA concentration and purity were determined using the NanoDrop2000c (Thermo Fisher Scientific. Reverse transcription of RNA into cDNA was accomplished with the PrimeScript RT reagent Kit with gDNA Eraser (RR047A, Takara, followed by quantitative RT-PCR (qRT-qPCR) using the TB Green® Premix Ex Taq™ (Tli RNaseH Plus) (RR420A, Takar(A). Data were normalized to GAPDH expression utilizing the 2^-ΔΔCT^ method. The standard deviation (SD) of samples carried out in technical triplicates was represented by error bars. The supplemental materials contain the primers used for RT-qPCR.

### Cell culture and scratch test

Since TLR9 expression is primarily documented in monocytes-macrophages in existing literature, we utilized TRAIL-resistant human acute myeloid leukemia cells (THP-1) for our research. THP-1 cells were obtained from the cell library of the Chinese Academy of Sciences Cells after undergoing multiple tests including mycoplasma detection, endotoxin testing, and DNA fingerprinting. The THP-1 cells were cultured in RPMI medium supplemented with 10% FBS, 100 U/mL penicillin, and 100 mg/mL streptomycin at 37 °C in a 5% CO_2_ incubator. Scratch assays were employed on densely adherent M0-THP1 cells to simulate tissue injury, following the method used by Nelson et al. [7]. To induce differentiation of THP-1 cells into a macrophage-like phenotype, 106 THP-1 cells were transferred to each of the six-well plates and cultured with 100 ng/mL phorbol 12-myristate 13-acetate (PMA) (P8139, Sigma-Aldrich) for 24 h. Eight scratches were made in cells within each well.

Human HaCaT cell lines were obtained from the cell library of the Chinese Academy of Sciences and cultured in RPMI medium supplemented with 10% FBS, 100 U/mL penicillin, and 100 mg/mL streptomycin at 37 °C in a 5% CO_2_ incubator. The influence of AREG was studied by adding different concentrations of recombinant AREG protein (100, 500, 1000 ng/mL) (P15514, novoprotein) to the culture medium for 48 h.

### Extraction and copy number determination of cell-free mtDNA

To detect the copy numbers of mtDNA, the DNeasy Blood & Tissue Kit (QIAGEN) was utilized following the manufacturer’s instructions to isolate the cell-free mtDNA in plasma and cell supernatant. Quantitative real-time PCR analyses were conducted to determine the mtDNA copy number in samples, using plasmids expressing mouse or human Cytochrome (CytB) with known copy numbers as standards. The mouse or human CytB sequences were inserted into the pUC57 plasmid, transformed and amplified in *Escherichia coli* (*E. coli*). The recombinant plasmid DNA was isolated from the bacteria and sequenced to confirm the sequences and acquire the DNA mass. The copy numbers of plasmids were determined by the formula: (6.02 × 10^23^) (OD26050)/(base number6601014) = copies/μL. Standard plasmids with concentrations of 100, 10, 1, 10^−1^, 10^−2^, 10^−3^, 10^−4^, 10^−5^, 10^−6^ ng/μL were diluted from the plasmids. The samples with standard substances were subjected to RT-PCR simultaneously. The CT-copy number standard curve was calculated using the data of standard plasmids, and the copy numbers of samples were calculated using the same formula.

### Extraction and application of mtDNA

The Mitochondrial DNA isolation kit (ab65321) was used according to the manufacturer’s instructions to extract mtDNA from mouse liver for injection into wound beds. The concentration and purity of mtDNA were determined using the NanoDrop2000c and stored at −20 °C. For injection into wound beds, 20 μg (mtDNA^lo^_LW) and 80 μg (mtDNA^hi^_LW) of mtDNA were used at PWD 0, 3, and 6. For in vitro tests, 100 ng/mL mtDNA was added to the cell culture medium for 24 h.

### Bulk-RNA sequencing

Bulk-tissue RNA sequencing was conducted using three to four replicates of mice skin wounds in each group using standard methods to make sure samples were strictly controlled for quality. The Illumina sequencing of the libraries was performed. Through z-transformation of fragments per kilobase of transcript per million mapped reads of the selected gene, gene expression was analyzed. Differential expression analysis of two groups (three biological replicates per group) was performed using the DESeq2 R package (v1.32.0). A *p* value < 0.05 and |log2(foldchange)| > 1.5 were set as thresholds for significant differential expression. We used the cluster Profiler R package to test the statistical enrichment of marker genes in KEGG and GO pathways.

#### Single cell RNA sequencing

##### Tissue dissociation

For scRNA-seq analysis, we collected five fresh samples at 2 days post scab detachment (SD2) per group. The wound tissues underwent enzymatic epidermal-dermal separation using the Epidermis Dissociation Kit (Epidermis Dissociation Kit, mouse; Miltenyi Biotec followed by dissociation of the epidermis and dermis parts. The resulting cells were then filtered, centrifuged, and resuspended in phosphate-buffered saline (PBS) with 0.5% bovine serum albumin (BSA). The dermis part was further dissociated using a mixed enzyme solution containing type I collagenase and trypsin, digested, and then processed similarly to the epidermis part. Subsequently, the dermis cells were combined with the epidermis cells after being subjected to red blood cell lysis buffer and removal of dead cells and debris by Dead Cell Removal MicroBeads (Miltenyi).

### Sequencing and data processing

Single-cell suspensions were used for Single-Cell RNA-seq (10x Genomics Chromium Single Cell Kit), followed by sequencing using an Illumina 1.9 mode. Subsequently, the reads were aligned, and expression matrices were generated using the Cell Ranger pipeline software. Downstream computational analysis involved merging different samples into one Seurat object using the RunHarmony function and performing filtering, normalization, scaling, canonical correlation analysis, principal component analysis, and dimensionality reduction using various R packages. Unsupervised clustering and differential gene expression analysis were carried out, followed by gene set enrichment analysis and receptor-ligand probability prediction among cell subpopulations.

### Pseudotime analysis

We utilized Monocle 2 for pseudo-temporal trajectory analysis to elucidate cell differentiation trajectories. These sophisticated algorithms position cells along a trajectory that corresponds to a specific biological process, such as cell differentiation, leveraging the distinct asynchronous progression of individual cells within an unsupervised framework [67, 68]. In the analysis, raw count data of highly variable genes, identified using the FindVariableGenes function from the Seurat package (with parameter y.cutoff = 0.5), were employed for pseudo-temporal trajectory analysis.

### RNA velocity analysis

ScVelo package was employed to perform RNA velocity analysis, which allowed for the identification of transient cellular states and prediction of directional progression of transcriptomic signatures along developmental trajectories. This analysis was based on gene-specific rates of transcription, splicing, and degradation of mRNA, with the results projected as a stream of arrows on the UMAP Embedding.

### Gene signature scoring based on scRNA-seq data

In the assessment of module scores and enrichment fractions for EGF pathway genes and cell cycle related genes’ expression in individual cells, the scRNA-seq data underwent AddModuleScore() computation. The hallmark genes, associated with the EGF or cell cycle in GO terms, were obtained from the Molecular Signatures Database (http://www.gsea-msigdb.org/gsea/msigdb/index.jsp) and utilized as the gene signature for scoring.

### Histopathology, Immunohistochemistry, and immunofluorescence microscopy

For histopathology, immunohistochemistry, and immunofluorescence microscopy, the samples were fixed with 4% paraformaldehyde and underwent ethanol and xylene dehydration at least 24 h beforehand. H&E staining and Masson’s trichrome staining were performed to observe re-epithelialization and collagen fiber deposition. In addition, frozen sections were collected for antibodies that can only be stained for IF-Fr. The primary antibodies used for immunofluorescence staining were as follows: TLR9 (Abcam, Ab134368, 1:150), IRF7 (sc-74472, Santa Cruz Biotechnology, 1:133), EEA1 (C45B10, Cell signaling technology, 1:133), CD3 (14-0032-82, Thermo Fisher Scientific, 1:100), Ki67 (Servicebio, GB121141, 1:100), TCR γ/δ (118101, Biolegend, 1:150), Goat Anti-Armenian hamster IgG H&L (Alexa Fluor® 647) (ab173004), CCL2 (26161-1-AP, Proteintech, 1:150), and AREG (16036-1-AP, Proteintech, 1:200). Biopsy sections were de-paraffinized and underwent antigen retrieval using Target Retrieval Solution. After washing and permeabilization with Tris-buffered saline (Quality Biological, 351-086-101) and 0.1% Tween 20 (Sigma, P2287) (TBST) buffer, the sections were blocked with 5% sheep serum and 1% Bovine Serum Albumin (BSA) (Fisher Bioreagents, BP9703-100) at room temperature for 1 h. Subsequently, the sections were incubated with the primary antibodies (as mentioned above) diluted in Antibody Diluent (Agilent Dako, S0809) overnight at 4 °C. After washing with TBST, the sections were incubated with fluorescent binding secondary antibodies at room temperature for 1 h. Following a final wash, the cell nuclei were stained with DAPI, and the sections were mounted with mounting medium. Immunofluorescent images were analyzed using DFC365FX (Leica or Olympus FV3000 Confocal Laser Scanning Microscope and processed with FIJI/ImageJ (National Institutes of Health, Bethesda, MD).

### Western blot analysis

To perform western blot analysis, protein lysates were extracted from the wound bed of three different types of snap-frozen skin tissue: WT_LW, TLR9agonist_LW, and WT_eLW. The proteins (15 µg per lane) were then separated using sodium dodecyl sulfate (SDS)-polyacrylamide gels and transferred to polyvinylidene fluoride membranes. After being blocked with 5% BSA, the membranes were incubated overnight with primary antibodies obtained from Cell Signaling Technology, Inc. (Danvers, MA), which included CCL2 (26161-1-AP, Proteintech, 1:500), AREG (16036-1-AP, Proteintech,1:400), IL-17A (sc-374218, Santa Cruz, 1:500), and GAPDH antibody (sc47724, Santa Cruz, 1:5000). The membrane was then washed and incubated with secondary horse radish peroxidase-labeled antibody. Bands were visualized using FluorChem E (ProteinSimple, San Jose, CA). Densitometry graphs were obtained by quantifying the phosphoprotein and total protein bands through densitometry with the use of Image-Pro Plus software (Media Cybernetics, Inc., Rockville, MD. Full and uncropped western blots were present in the supplemental materials.

### ALP staining

Whole-mount HFN assay to detect ALP+ dermal papilla (DP) was performed following the protocols in [4].

### Flow cytometry analysis

For flow cytometry analysis, single cells were digested from skin wounds and pre-incubated with purified anti-CD16/CD32 antibody (101301, BioLegend (1.0 μg per 106 cells in 100 μl volume) for 5–10 min to block Fc receptors. The cell suspensions were then co-incubated with fixable viability dye (eFluor™ 780, 65-0865-14, eBioscience) and antibodies against surface markers CD45(PE/Cy7, 147703, BioLegend, Cd3e(APC, 100311, BioLegend, CD4(FITC, 11-0041-82, eBioscience), CD8a(Alexa Fluor™ 700,56-0081-82, eBioscience), and TCRγ/δ(eflour-450,48-5711-82, eBioscience) at 1:400 dilution for 30 min at 4 °C in the dark (100 μl per antibody per sample). After fixation and permeabilization, cells were incubated with antibodies against intracellular marker IL-17A (PE,12-7177-81, eBioscience) at 1:400 dilution for 30 min at 4 °C in the dark (100 μl per antibody per sample). Fluorescence Minus One (FMO) groups were applied in every test. Flow cytometry analysis was performed using Attune Nxt flow cytometer (Thermo Fisher Scientific and FlowJo (v10.8.1). The experiments were performed independently three times (*n* = 3).

### Statistics and reproducibility

Statistical analyses were performed with Case Viewer software, Image J software, and Prism 9.0 software using two-tailed *t*-tests/ *t*′-test or one-way analysis of variance (ANOVA)with Tukey post-hoc test. Prior to performing t-tests or ANOVA, distributional checks were conducted to ensure normality. For multiple group comparisons, a test of homogeneity of Variance was conducted, and ANOVA analysis was applied only after confirming similar variances. Data were presented as mean ± standard deviation, and a p-value less than 0.05 was considered statistically significant (*p < 0.05, **p < 0.01, ***p < 0.001, ****p < 0.0001), while ns indicates no statistically significant difference.

For normally distributed data, outliers were primarily identified by excluding values that deviated more than 3 standard deviations from the mean. For non-normally distributed data, outliers were determined using a box plot approach, where values below (Q1 − 1.5IQR) or above (Q3 + 1.5IQR) were considered outliers. These criteria were established based on standard statistical analysis practices.

Random group assignment was conducted using a randomized design. Animals were initially ordered by their original body weight from smallest to largest, then random numbers were generated from a random number table to determine group allocation.

Blinding procedures were implemented as follows: each animal was assigned a unique numeric code with concealable group information during model construction and grouping. During data collection, information was gathered while concealing group details. Subsequently, data analysis and processing were performed by another researcher who was also blinded to the group allocations.

### Supplementary information

Supplemental file1-figures and tables

Supplemental file2-uncropped gels

## Data Availability

The sequencing data in this study were deposited in the NCBI Gene Expression Ominbus (GEO) under accession number GSE272509. All other data supporting the findings of this study are available within the article and its supplementary files. Any additional requests for information can be directed to, and will be fulfilled by, the corresponding authors.
