# Supplementary material for: TLR9 activation in large wound induces tissue repair and hair follicle regeneration via γδT cells
Source: Cell Death Dis. 2024 Aug 17;15(8):598. doi: 10.1038/s41419-024-06994-y (PMC11330466; doi:10.1038/s41419-024-06994-y)
Supplement: Supplementary file 1 — Supplemental file1-figures and tables [file 41419_2024_6994_MOESM1_ESM.docx]

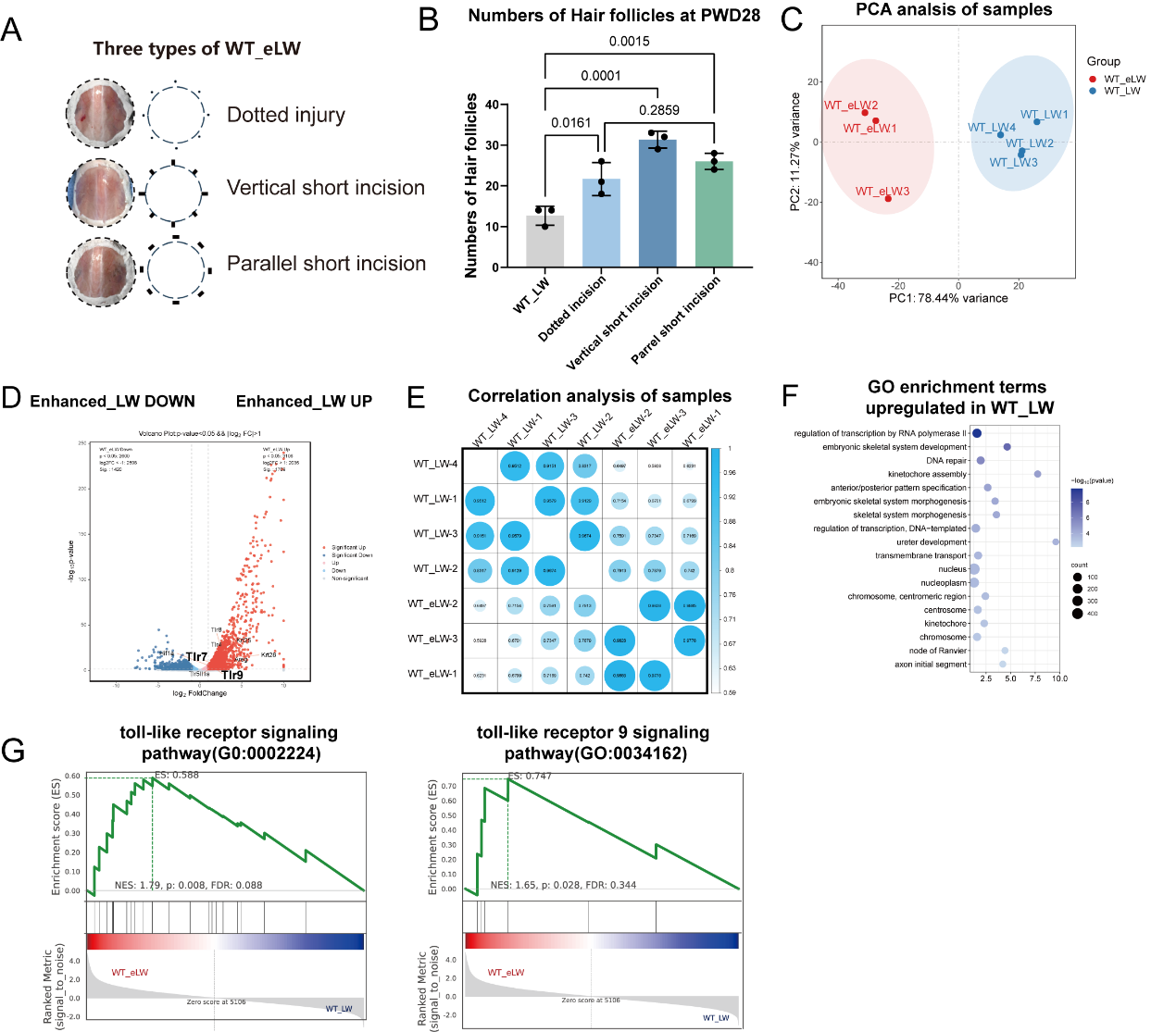


**Fig S1. Evaluation of the wound healing process with WT_eLW.** **A)** The three types of WT_eLW and the wound models. **B)** Number of regenerated hair follicles in PWD28. Statistical analysis was performed using one-way ANOVA with Dunnett’s multiple comparisons test. n=3 for each group. **C)** The PCA analysis of bulk-RNA sequence samples. **D)** The volcano plot showing the genes expressed in WT_eLW and WT_LW. The threshold for screening differential genes was p-value<0.05 and |log2FC|>1. **E)** Correlation analysis heatmaps of WT_eLW and WT_LW. **F)** GO terms enriched in WT_LW. **G)** The GSEA enrichment analysis of toll-like receptor signaling pathway and toll-like receptor 9 signaling pathway. All error bars ±SD.


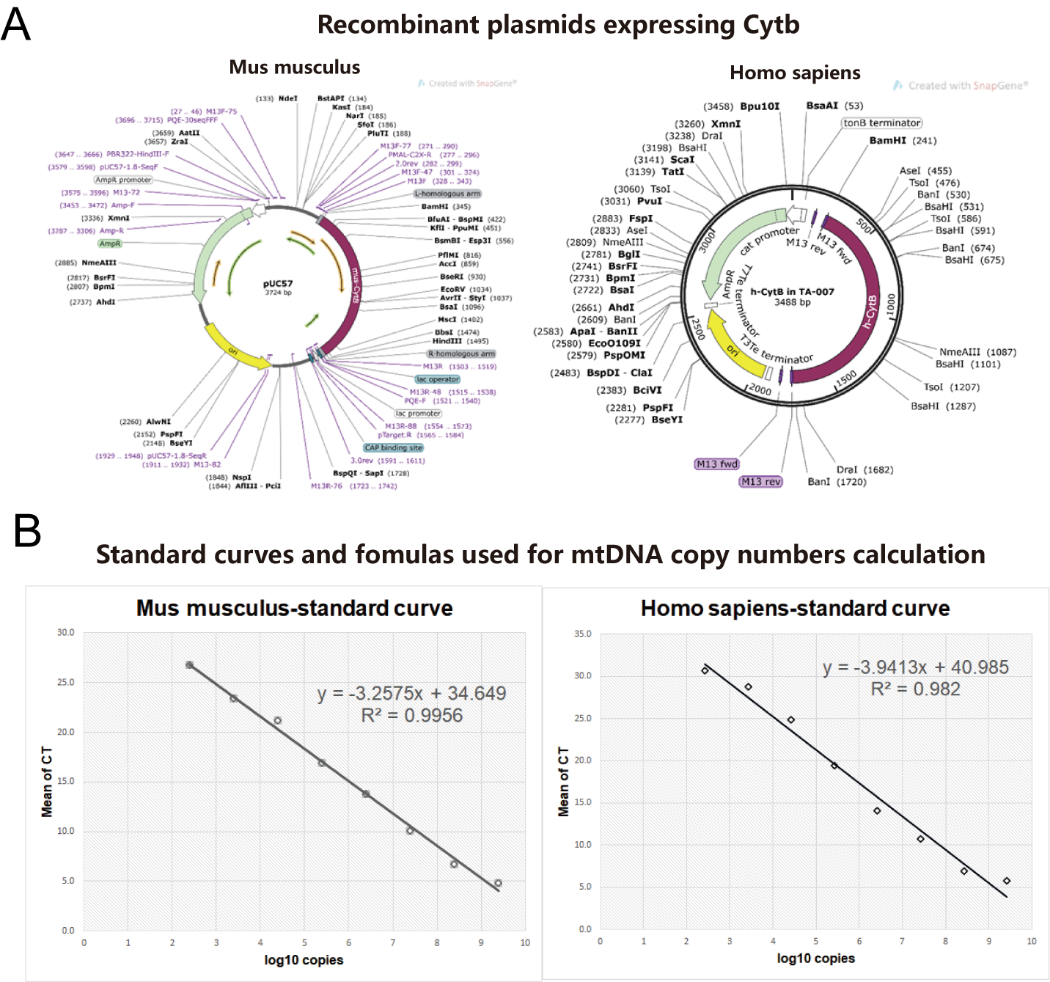


**Fig S2. A)** The recombiant plasmids expressing cytb of mus musculus and homo sapiens, respectively. **B)** Standard curves and formulas between CT number and copy numbers calculated by plasmids standards.


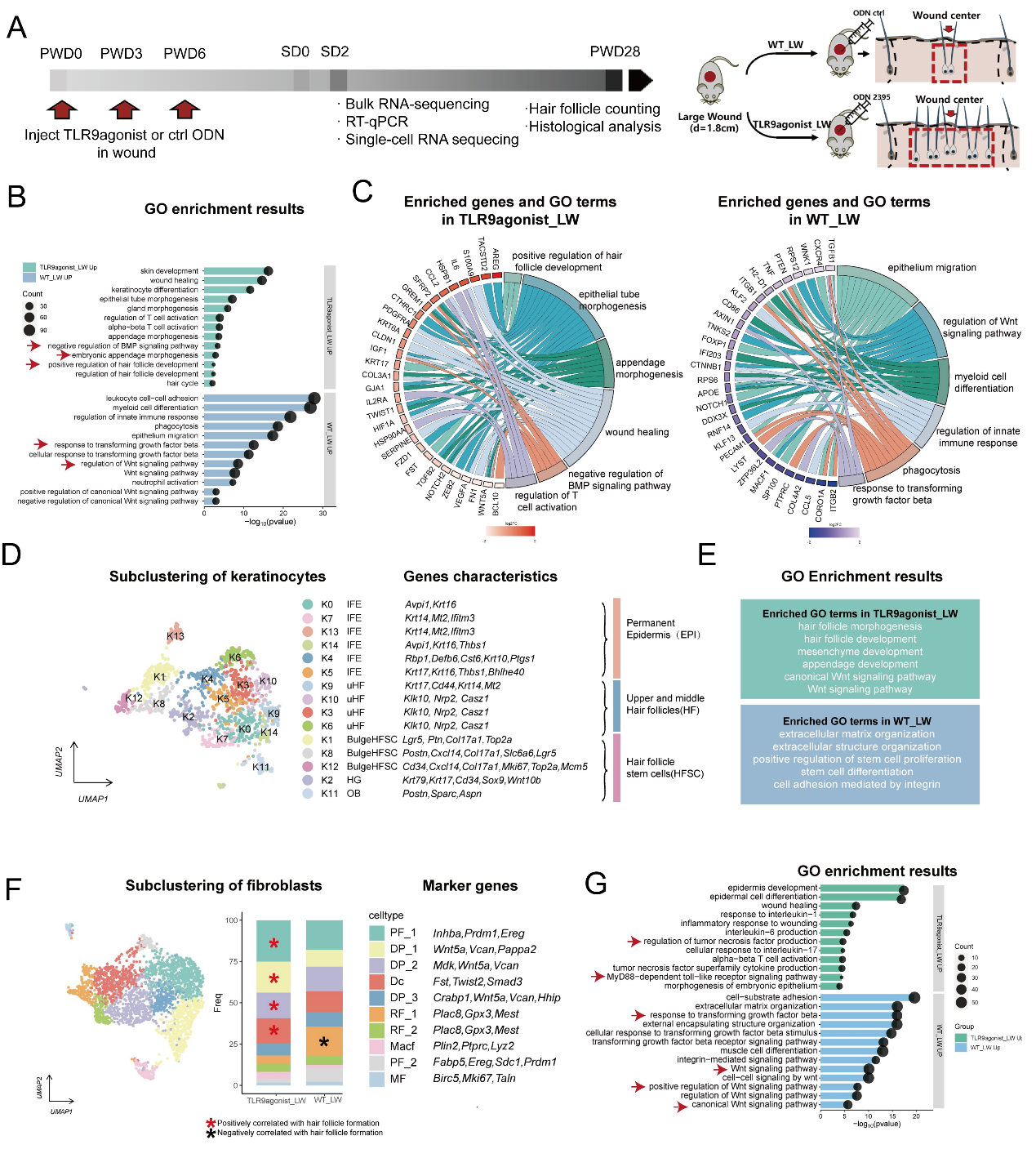


**Fig S3. The subcluster and analysis of neutrophils and macrophages. A)** Workflow for evaluating the function of TLR9 and mtDNA in large-scale wound healing. The second day after scab detachment (SD2) was chosen as our observation time for hair follicle neogenesis. **B)** The subclustering of neutrophils and relative cell ratios and marker genes. **C)** The subclustering of macrophages and dendritic cells. **D)** The annotations and marker genes of macrophages and dendritic cells. **E)** The KEGG enrichment terms for every subclusters. **F)** The expression of phagocytosis related genes in macrophages and dendritic cells. And the genes in wnt pathway of all cells and fibroblasts. **G)** The expression of inhibitors of wnt pathway in all cells.


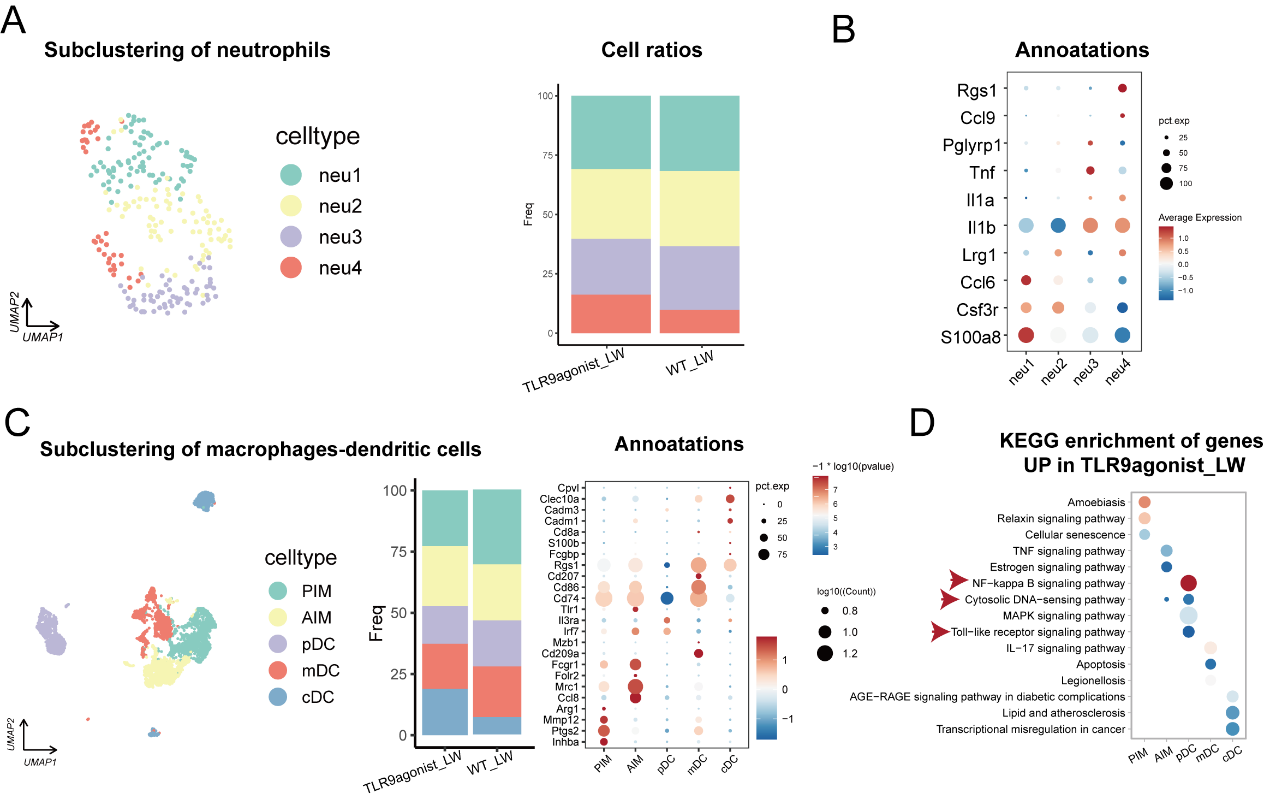


**Fig S4. The subcluster and analysis of neutrophils and macrophages. A)** The subclustering of neutrophils and relative cell ratios. B) Marker genes used for annotations of neutrophils. **C)** The subclustering, annotations and marker genes of macrophages and dendritic cells. **D)** The KEGG enrichment terms for every subclusters.


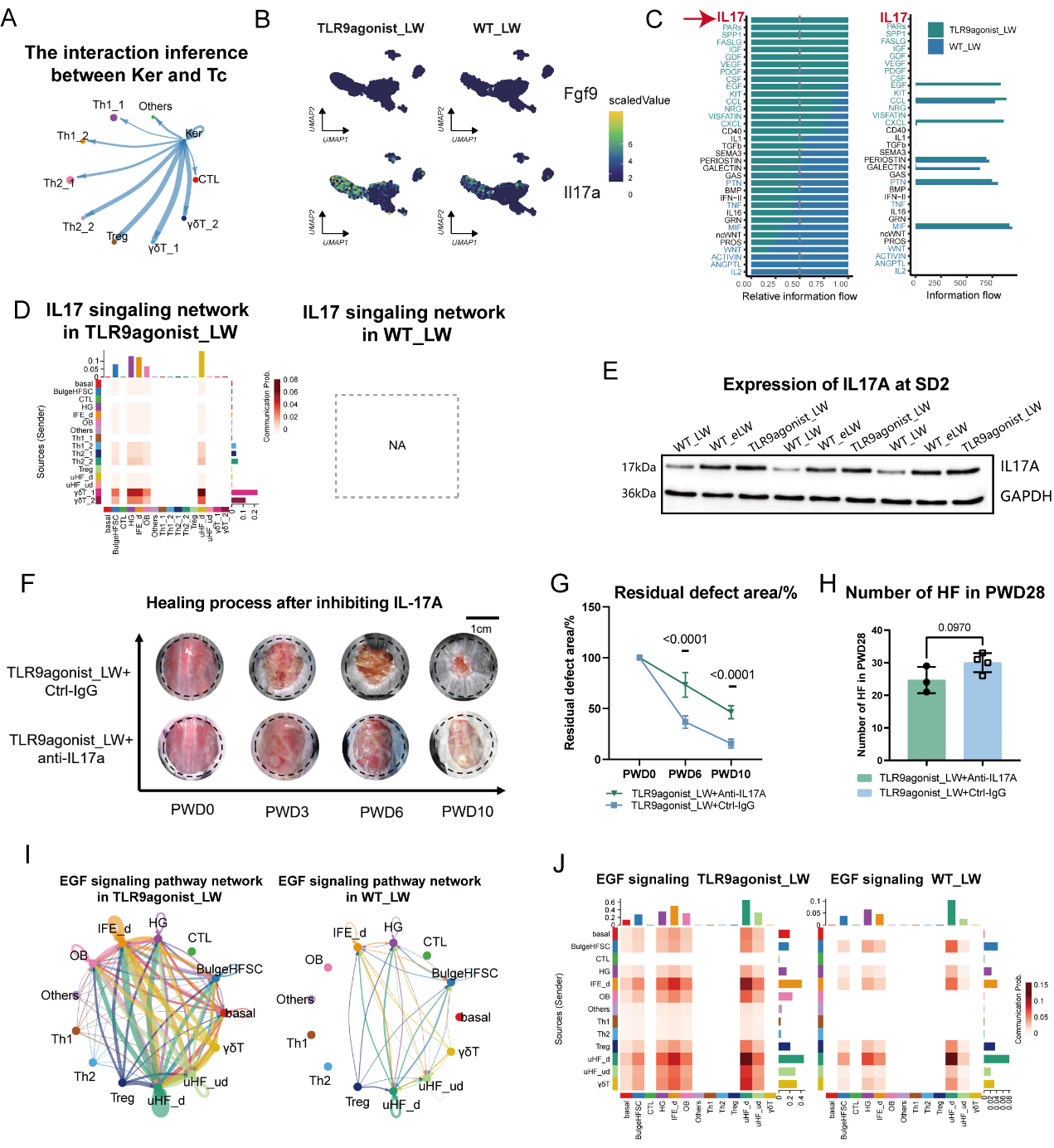


**Fig S5. The function of γδT in promoting WIHN is not dependent on IL-17A. A)** The chord graph showing the interaction inference between Ker (keratinocytes) and Tc (T cells). **B)** The expression of Fgf9 and IL-17a in scRNA data. The feature plots showing the expression of IL-17a in T cells were mainly in γδT cells and the expression levels were different in TLR9agonist_LW and WT_LW. **C)** The differential interaction pathways in TLR9agonist_LW and WT_LW. **D)** The IL-17 interaction networks between T cells and keratinocytes in TLR9agonist_LW and WT_LW. **E)** The expression of IL-17A in WT_LW, WT_eLW and TLR9agonist_LW at SD2 was detected by western blotting. GAPDH was used as loading control. **F)** The photos of wounds in TLR9agonist_LW injected with Ctrl-IgG (30000-0-AP, Proteintech), or anti-IL-17A (10μg per time per mouse. PAB30184, Bioswamp). Pictures were taken from same mice in the time span. **G-H)** The changes of healing speeds and regeneration outcomes after injection of anti-IL-17A or Control-IgG into the wound bed at PWD 0,3,6. n=3-4 for each group. **I)** The chord plots showing the EGF signaling pathway network between T cells and keratinocytes in TLR9agonist_LW and WT_LW. The interaction of EGF pathway between T cells and keratinocytes was mostly between γδT cells to uHF_d, IFE_d, OB and HG. **J)** The heatmaps showing the EGF signaling pathway network between T cells and keratinocytes in TLR9agonist_LW and WT_LW.


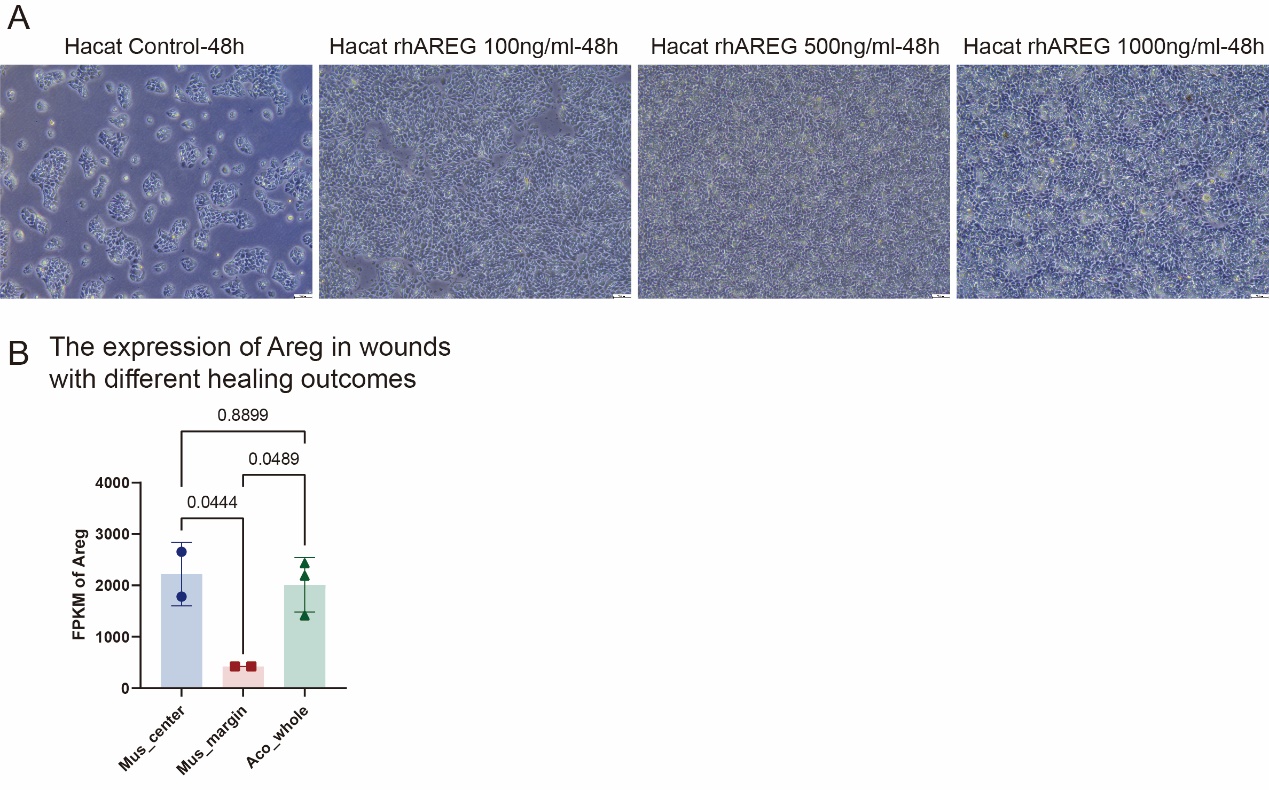


**Fig S6. The effect of recombinant AREG to Hacat after 48h. A)** The differential appearance of cells growth after being cultured with mouse recombinant AREG protein (100ng/mL, 500ng/mL, 1000ng/mL) (P15514, novoprotein) in the culture medium for 48h. **B)** The FPKM (fragments per kilobase of transcript per million fragments) of Areg in wounds with different WIHN outcomes from GSE159939. The wound center at PWD14 in Mus musculus (high-WIHN) and the whole wound at PWD14 in Acomys spiny mice (high-WIHN) have higher expression level of Areg than the wound margin at PWD14 in Mus musculus (low-WIHN).

**Table S1: Primers used for RT-qPCR:**

| Gene symbol | Species | Forward (5’-3’) | Reverse (3’-5’) |
| --- | --- | --- | --- |
| Cytb | Mouse | TGAGGGGGCTTCTCAGTAGA | CTGTTTCGTGGAGGAAGAGG |
| Cytb | Human | ATGACCCCAATACGCAAAAT | CGAAGTTTCATCATGCGGAG |
| Tlr9 | Mouse | TGTGAGCTGAAGCCTCATGG | GGACAGGTGGACGAAGTCAG |
| Tlr9 | Human | CTGCGTTTTGTCGAAGACCA | CCCACCTGTCACTCAAGTACA |
| Areg | Human | TGAGATGTCTTCAGGGAGTG | AGCCAGGTATTTGTGGTTCG |
| Wnt10b | Human | CATCCAGGCACGAATGCGA | CGGTTGTGGGTATCAATGAAGA |
| Ctnnb1 | Human | CATGCACCTTTGCGTGAGCA | CCCCCTCCACAAATTGCTGC |
| Twist1 | Human | GTCCGCAGTCTTACGAG | CACGCCCTGTTTCTTTG |
| Twist2 | Human | AGAAGTCGAGCGAAGATG | CCTGGTAGAGGAAGTCTATG |
| Bmp6 | Human | CAGCCTGCAGGAAGCATGAG | CAAAGTAAAGAACCGAGATG |
| β-actin | Mouse/Human | CCAACCGTGAAAAGATGACC | ACCAGAGGCATACGGGACA |
