# Supplementary figures and images for: TLR9 activation in large wound induces tissue repair and hair follicle regeneration via γδT cells
Source: Cell Death Dis. 2024 Aug 17;15(8):598. doi: 10.1038/s41419-024-06994-y (PMC11330466; doi:10.1038/s41419-024-06994-y)

Full unedited blot/gel for Figure 4J


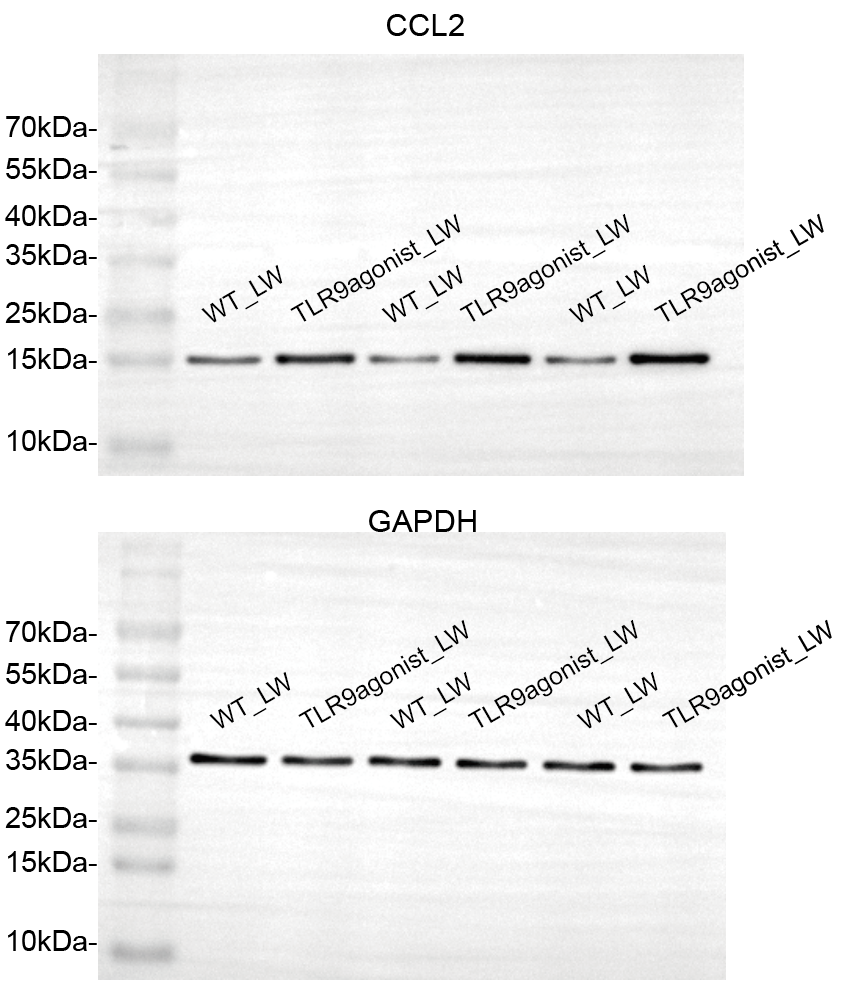


Full unedited blot/gel for Figure S5E


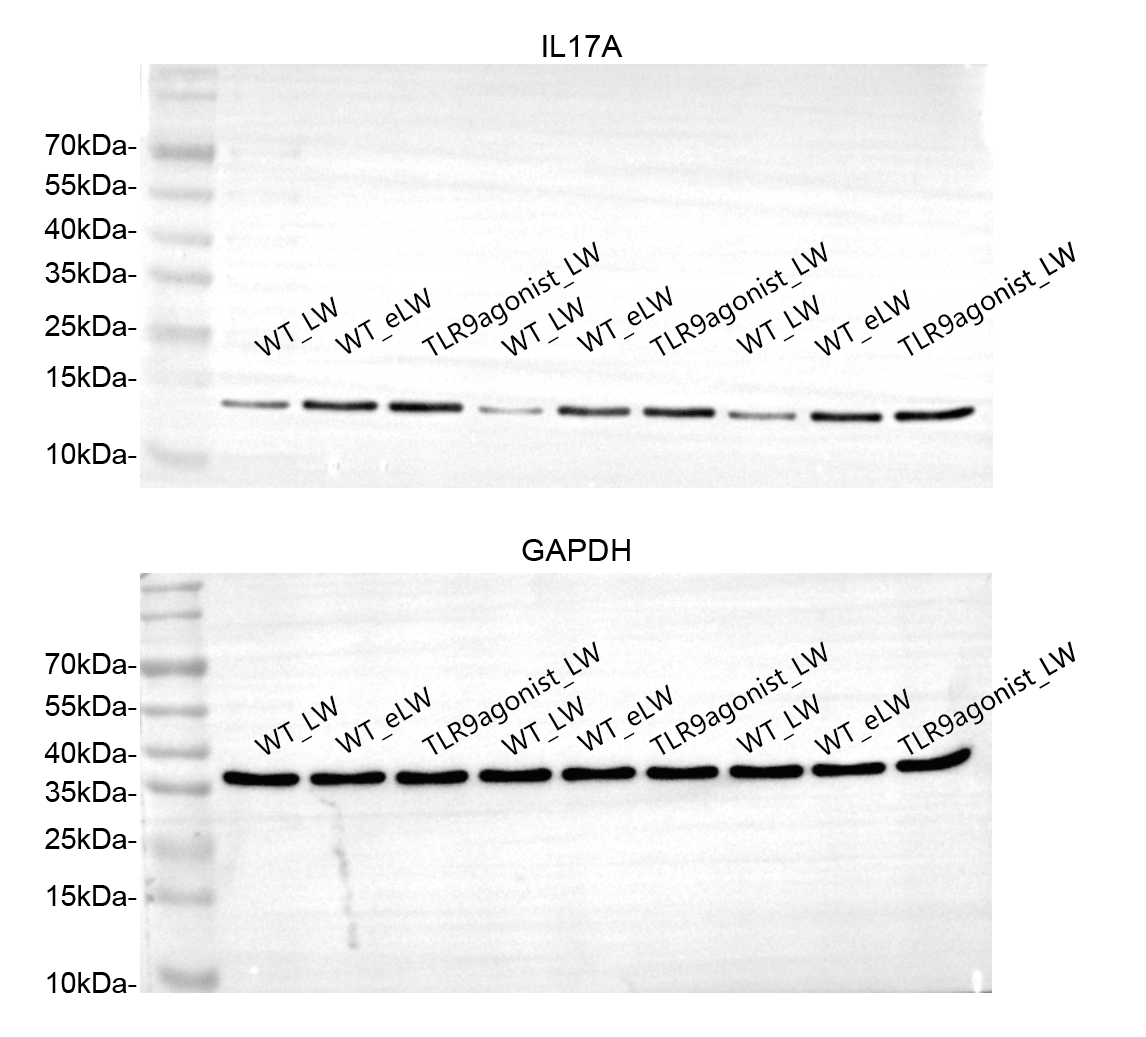

Supplement: Supplementary file 2 — Supplemental file2-uncropped gels [file 41419_2024_6994_MOESM2_ESM.docx]
